# Infection with the Makona variant results in a delayed and distinct host immune response compared to previous Ebola virus variants

**DOI:** 10.1038/s41598-017-09963-y

**Published:** 2017-08-29

**Authors:** Krista Versteeg, Andrea R. Menicucci, Courtney Woolsey, Chad E. Mire, Joan B. Geisbert, Robert W. Cross, Krystle N. Agans, Daniel Jeske, Ilhem Messaoudi, Thomas W. Geisbert

**Affiliations:** 1 Galveston National Laboratory, Galveston, TX USA; 2 Department of Microbiology and Immunology, University of Texas Medical Branch, Galveston, TX USA; 3 Division of Biomedical Sciences, University of California-Riverside, Riverside, CA USA; 4 Department of Molecular Biology and Biochemistry, University of California-Irvine, Irvine, CA USA

**Keywords:** RNA sequencing, Viral infection

## Abstract

*Zaire Ebolavirus* (ZEBOV) continues to pose a significant threat to human health as highlighted by the recent epidemic that originated in West Africa and the ongoing outbreak in the Democratic Republic of the Congo. Although the ZEBOV variant responsible for this epidemic (Makona) shares significant genetic similarity with previously identified variants (Kikwit and Mayinga), recent reports suggest slower disease progression in nonhuman primates. However, the pathogenesis caused by the new variant is not fully understood. We present the first comprehensive approach in understanding ZEBOV-Makona pathogenesis in cynomolgus macaques by measuring changes in immune cell frequencies, plasma levels of immune mediators, and differentially expressed genes (DEGs) within whole blood (WB) and peripheral blood mononuclear cells (PBMC). Our combined approach revealed a link between: 1) increased interferon-stimulated gene expression, IFNα levels, and activated plasmacytoid dendritic cells; 2) higher proinflammatory gene expression, cytokine and chemokine levels, and non-classical monocytes; 3) gene signature of leukocyte activation and increased granulocytes; and 4) decreased expression of lymphocyte related genes and lymphopenia. In addition, our data strongly indicate delayed disease progression as well as limited overlap (~30%) in host transcriptome changes following ZEBOV-Makona infection compared to ZEBOV-Kikwit. These observations provide novel insight into the molecular mechanisms of ZEBOV-Makona pathogenesis.

## Introduction

*Zaire Ebolavirus* (ZEBOV) is a member of the *Filovirus* family with a single strand, negative sense RNA genome encoding 9 viral proteins^1^. ZEBOV infection in humans is characterized by hemorrhage, lymphopenia, high levels of circulating pro-inflammatory mediators, liver failure, and disseminated intravascular coagulation, which culminate in death due to hypovolemic shock and multi-organ failure^2–4^. The traditionally remote locations and small magnitudes of ZEBOV outbreaks have precluded in-depth studies of ZEBOV pathogenesis in humans. Therefore, different animal models have been used to elucidate disease progression and host responses to ZEBOV. Despite the experimental advantages that rodents offer, the need for adapted EBOV strains is a major limitation. Recently, a ferret model for EBOV infection has been described in which wild-type virus induces uniform lethality featuring many hallmark features of EBOV pathogenesis^5^; however, the current dearth of reagents available to study immune parameters limits the utility of this model to study immune responses. In contrast, infection of nonhuman primates (NHP, rhesus and cynomolgus macaques) with wild-type ZEBOV variants results in lethal disease^6, 7^ with a similar presentation as humans^4^. Studies in NHP have demonstrated that ZEBOV initially infects monocytes and dendritic cells in draining lymph nodes before disseminating to the liver, adrenal gland, kidney, and lymphoid tissue^6^. In addition, consumption of clotting factors and high levels of fibrin degradation products contribute to the development of the characteristic petechial rash and hemorrhage from mucosal membranes. End-stage disease in macaques is typified by liver necrosis, loss of splenic structure, and lymphopenia^8, 9^.

In December of 2013, a ZEBOV outbreak was reported in Guinea that quickly spread to Sierra Leone and Liberia resulting in the first and largest ZEBOV epidemic. Ultimately, 10 countries were affected with over 28,600 cases and 11,300 fatalities^10^. The ZEBOV variant responsible for the West Africa epidemic was named Makona, after the river that borders Guinea, Liberia and Sierra Leone. Although the end of the epidemic was declared in January 2016, sporadic cases continued to occur in Guinea and Sierra Leone, possibly due to the persistence of the virus in immune privileged sites, such as the testes, eyes, and central nervous system.

Sequence analysis indicates that ZEBOV-Makona has a 97% nucleotide identity to Mayinga and Kikwit ZEBOV variants^11^. In addition to the symptoms characteristic of Ebola hemorrhagic fever (EHF), ZEBOV-Makona infection has been associated with more pronounced gastrointestinal symptoms (severe vomiting and diarrhea). Moreover, recent NHP studies show a delay in disease progression after infection with ZEBOV-Makona compared to ZEBOV-Mayinga^12^. However, little is known about the progression of disease caused by this newly identified variant. To address this gap in knowledge, we conducted a longitudinal study to characterize the host immune response to ZEBOV-Makona infection in NHP using a multipronged approach that combined immunological assays and next generation sequencing in both whole blood (WB) and peripheral blood mononuclear cells (PBMC). Our data show delayed appearance of clinical symptoms as well as overlapping but distinct host transcriptome changes during ZEBOV-Makona infection compared to ZEBOV-Kikwit in ZEBOV-Makona-infected animals thereby providing novel insight into ZEBOV-Makona pathogenesis.

## Results

### Disease signs correlate with viral replication

**Figure 1:**
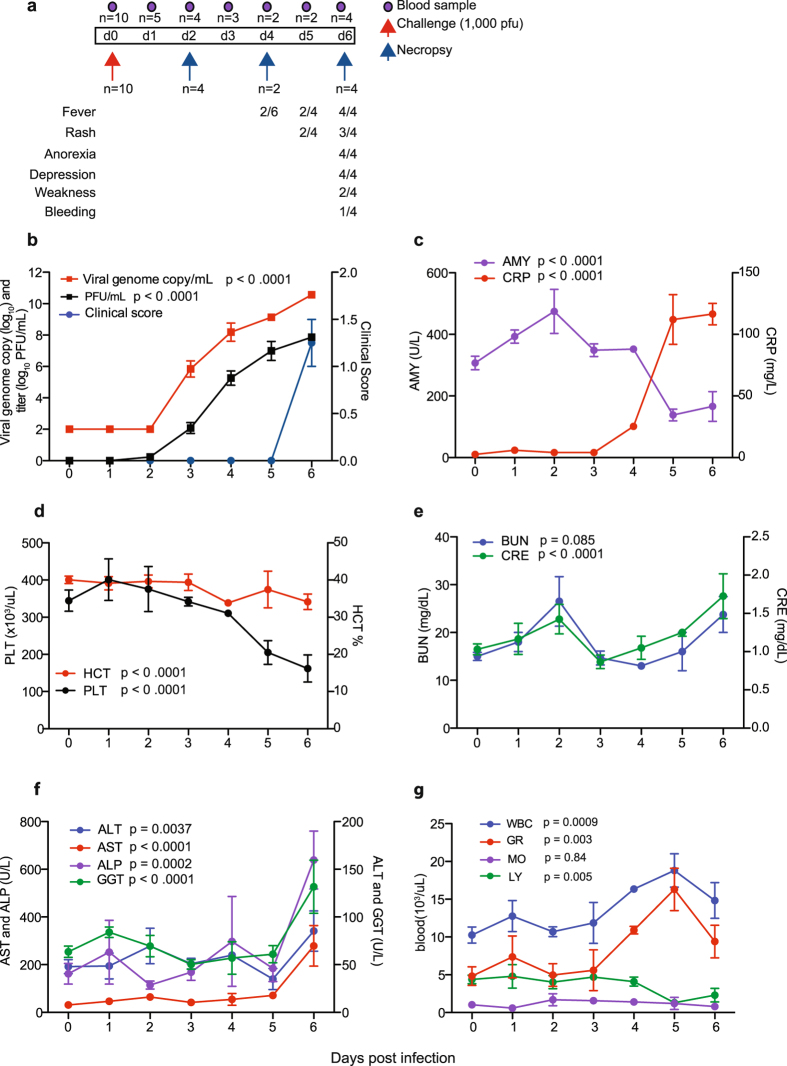
Disease signs correlate with viral replication. (**a**) Study time line and clinical observations. (**b**) Infectious virus was quantified by plaque assay on Vero cells and viral genome copies were measured using RT-qPCR with primers/probe targeting VP30. Average clinical scores as obtained by a scoring sheet (Supplementary Table S1). (**c**) Amylase (AMY) and C-reactive protein (CRP) plasma levels. (**d**) Percentage hematocrit and platelets counts. (**e**) Blood urea nitrogen (BUN) and creatinine (CRE) levels. (**f**) Alanine aminotransferase (ALT), Aspartate aminotransferase (AST), alkaline phosphatase (ALP), gamma-glutamyltransferase (GGT) levels. (**g**) White blood cells (WBC), lymphocyte (LY), monocyte (MO), and granulocyte (GR) counts throughout infection. A linear model was used to perform statistical analysis; p-values listed for each parameter represent overall effect throughout infection.

Ten male cynomolgus macaques were challenged with 1000 PFU of ZEBOV-Makona strain C07. We selected this isolate since it is one of the earliest and better characterized isolates from Guinea that was also used in a recent NHP study^12^. Fever (temperatures 2 °F higher than baseline) was evident on or after 4 days post infection (DPI). Anorexia and mild to moderate depression were noted 6 DPI in 4/4 animals, whereas mild petechial rashes on arms, chest, and groin regions was evident in 3/4 animals. Two animals on day 6 exhibited a hunched posture and general weakness and one monkey had rectal bleeding (Fig. 1a). However, these clinical signs became scorable only 6 DPI (Supplementary Table S1, Fig. 1b). Viral titers were measured in plasma by plaque assay and viral RNA was measured in whole blood using one-step RT-qPCR. Viremia was detected 3 DPI and significantly increased as infection progressed (Fig. 1b).

Levels of circulating C-reactive protein (CRP), indicative of inflammation, correlated with viremia, and were increased slightly 4 DPI, followed by a large increase at 5 and 6 DPI (Fig. 1c). Similarly, changes in blood amylase levels, characteristic of pancreatic injury, weren’t detected until 5-6 DPI, when they significantly decreased (Fig. 1c). Hematocrit and platelet numbers also decreased 5-6 DPI, which may be associated with coagulopathy and detrimental changes in microcirculation (Fig. 1d). Levels of blood creatinine (CRE), indicative of kidney function, as well as liver enzymes (alanine transaminase (ALT), aspartate transaminase (AST), alkaline phosphatase (ALP), and gamma-glutamyl transpeptidase (GGT)) significantly increased only 6 DPI when disease signs were more evident (Fig. 1e and f). Total white blood cell numbers (WBC) increased 4-5 DPI driven by a significant increase in granulocytes (neutrophils) before declining 6 DPI due to loss of both granulocytes and lymphocytes, while monocyte numbers remained relatively consistent throughout the study (Fig. 1g).

Changes in circulating immune mediators correlated with disease progression. In plasma, we detected significantly increased levels of inflammatory cytokines 6 DPI including IL-1β, IL-18, IFNγ, and IL-6 as well as regulatory cytokines IL-1RA and IL-4 (Supplementary Fig. S1a,b). We also observed a sharp increase in IFNα, a potent antiviral cytokine, 6 DPI (Supplementary Fig. S1b). Lymphocyte populations were also likely impacted by significant decreases in levels of IL-7, which plays a role in B and T-cell development and homeostasis, by 5-6 DPI (Supplementary Fig. S1b). Furthermore, ZEBOV-Makona infection resulted in upregulation of several chemokines 6 DPI (Supplementary Fig. S1c) including T-cell attractants, I-TAC (CXCL11) and MIG (CXCL9); leukocyte attractant, MIP1α; monocyte attractant, MCP-1; eosinophil attractant, eotaxin; and B-cell attractant, CXCL13. We also detected a significant decrease in growth factor PDGF-BB, which also coincides with characteristic thrombocytopenia seen in filovirus infection (Supplementary Fig. S1c).

### ZEBOV-Makona infection results in early activation of innate immune cells

**Figure 2:**
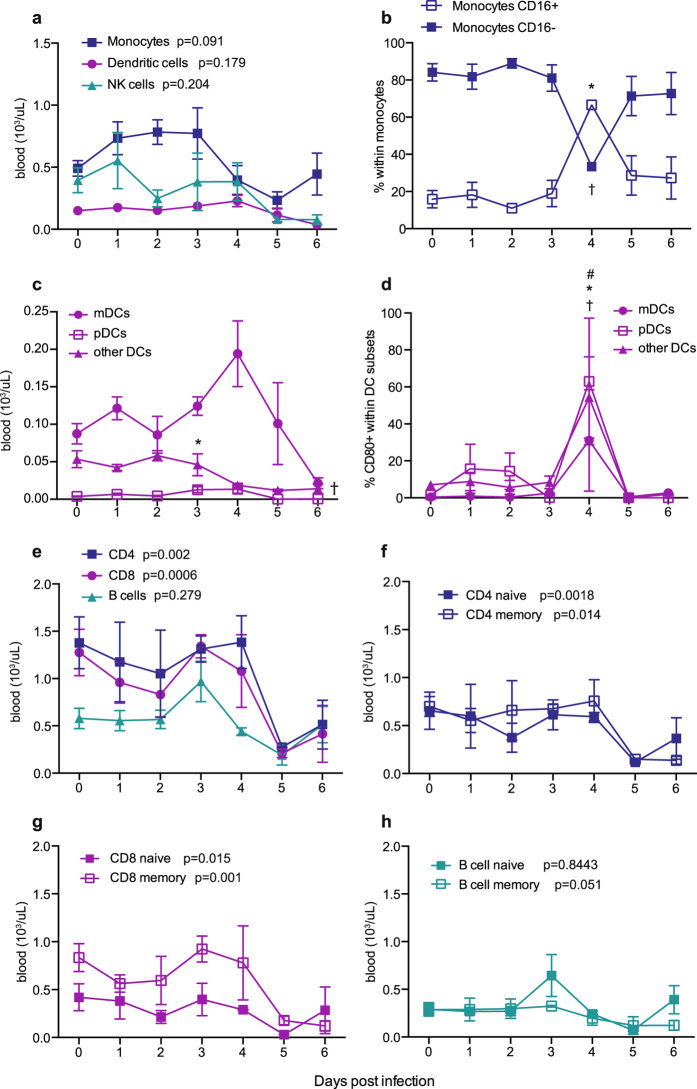
ZEBOV-Makona infection results in early activation of innate immune cells and lymphopenia. (**a**) Frequency of dendritic cells (DCs, CD14^−^HLA-DR^+^), monocytes (CD14^+^HLA-DR^+/−^), and NK cells (CD3^−^CD20^−^CD14^−^CD8a^+^) were measured by flow cytometry (FCM). (**b**) Frequency of classical (CD16^−^) and intermediate/non-classical (CD16^+^) monocytes. (**c**) Frequencies of myeloid DCs (mDC, CD123^−^CD11c^+^), plasmacytoid DCs (pDC, CD123^+^CD11c^−^) and other DCs (CD123^−^CD11c^−^). (**d**) Frequency of DC subsets expressing CD80. (**e**) Frequency of CD20^+^ B-cells, CD4^+^ and CD8^+^ T-cells. (**f**,**g**) Frequency of naïve (CD28^+^CD95^−^) and memory (CD28^+/−^CD95^+^) T-cells within CD4 (**f**) and CD8 (**g**) subsets. (**h**) Frequency of naïve (CD27^−^) and memory (CD27^+^) B-cells. For frequency changes in total monocyte(s), DCs, NK cells, CD4 T-cells, CD8 T-cells and B-cells, a linear model was used to perform statistical analysis; p-values listed for each parameter represent overall effect throughout infection. For changes in subset frequencies within monocytes, DC, and NK cells, a nonparametric trend where each time point is modeled by its own mean was assumed for statistical analysis; symbols (*,†,#) denote p-value ≤ 0.05 at the indicated time point compared to 0 DPI: ^*^for CD16^+^ Monocytes and pDCs; ^†^for CD16^−^ Monocytes and other DCs; ^#^for mDCs.

To characterize the immune response to ZEBOV-Makona, we used flow cytometry to measure changes in frequency and phenotype of circulating immune cells in PBMC (gating strategy described in Supplementary Fig. S2). Frequency of monocytes (defined as CD3^−^CD20^−^CD14^+^) remained relatively stable throughout infection with the exception of a small decrease 5 DPI (Fig. 2a). Further analysis indicates a transient yet significant increased frequency of intermediate/non-classical monocytes (CD16^+^) (p = 0.005) 4 DPI (Fig. 2b). This increased frequency of CD16^+^ monocytes returned to baseline values 6 DPI.

Total number of DCs (CD3^−^CD20^−^CD14^−^HLA-DR^+^) also remained stable with the exception of a small increase 4 DPI followed by a decline 5 DPI (Fig. 2a). We then analyzed changes in the three distinct DC subsets: myeloid dendritic cells (mDC; CD11c^+^CD123^−^), plasmacytoid dendritic cells (pDC; CD11c^−^CD123^+^), and other dendritic cells (CD11c^−^CD123^−^) (Fig. 2c). This analysis revealed that the increase in DC numbers 4 DPI was driven by a slight increase in the frequency of mDCs (p = 0.116) and a significant increase in pDCs 3 DPI (p = 0.0222) and 4 DPI (p = 0.0775). The increase in mDC and pDC numbers 4 DPI was accompanied by increased expression of the activation marker CD80 by all three DC subsets (Fig. 2d). The number of other DCs, in contrast, declined 6 DPI (p = 0.033) (Fig. 2c).

We detected a small decrease in the total number of natural killer (NK) cells (CD3^−^CD20^−^CD14^−^CD8α^+^) at the later stages of the disease 5-6 DPI (Fig. 2a). Although frequencies of cytotoxic NK cells (CD16^+^) and cytokine-producing NK cells (CD16^−^) remained stable throughout infection (Supplementary Fig. S3a), the frequency of cytokine-producing NK cells expressing CD159 was transiently increased 1 DPI (p = 0.0072) (Supplementary Fig. S3b).

### ZEBOV-Makona infection results in lymphopenia

PBMC were also analyzed by flow cytometry to determine changes in frequency of circulating lymphocytes (Fig. 2e–h). CD4^+^ T-cells (CD3^+^CD20^−^CD4^+^) and CD8^+^ T-cells (CD3^+^CD20^−^CD8^+^) were classified into naïve (CD28^+^CD95^−^) and memory (CD28^+/−^CD95^+^) subsets (Fig. 2f,g). Similarly, B-cells (CD3^−^CD20^+^) were also classified as naïve (CD27^−^) or memory (CD27^+^) (Fig. 2h). Overall, T-cell numbers, both memory and naïve, declined 5 and 6 DPI (Fig. 2e–g). Although the number of total B-cells remained stable, memory B-cells significantly decreased 5-6 DPI (Fig. 2h). In addition to analyzing frequency of T and B-cell subsets, we assessed proliferation by measuring changes in the expression of Ki67, a nuclear protein associated with DNA replication. Memory CD4^+^ and CD8^+^ T-cells were further divided into three groups: central memory (CM; CD28^+^CD95^+^CCR7^+^), effector memory (EM; CD28^−^CD95^+^CCR7^−^) and transitional effector memory (TEM; CD28^+^CD95^+^CCR7^−^). In contrast to lymphocyte numbers, the proliferation of memory CD4^+^ and CD8^+^ T-cell subsets significantly increased 4-6 DPI while that of naïve T-cells remained unchanged (Supplementary Fig. S3c,d).

### ZEBOV-Makona induces early and sustained upregulation of innate immune genes

**Figure 3:**
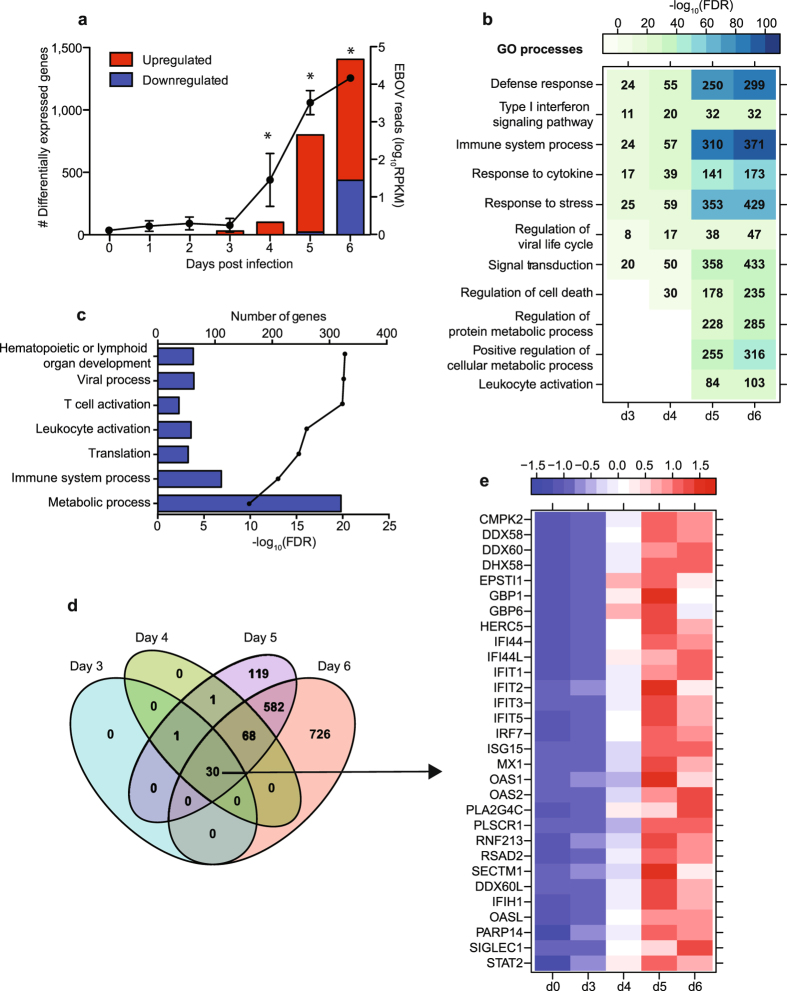
ZEBOV-Makona infection induces sustained large transcriptional changes of innate immune genes. (**a**) Bar graph depicts number of protein-coding differentially expressed genes (DEGs; defined as those ≥log2 fold change compared to 0 DPI and FDR-corrected p-value ≤ 0.05) that have human homologues. Line graph indicates number of viral transcripts reported as normalized by reads per kilobase per million mapped (RPKM); the *EdgeR* package was used to determine statistically significant changes in viral reads; *denote p-value ≤ 0.05 at the indicated time point compared to 0 DPI. (**b**) Heatmap representing functional enrichment of DEGs upregulated 3-6 DPI; color intensity represents the statistical significance (shown as −log_10_ of the FDR-corrected p-value); range of colors is based on the lowest and highest −log_10_(FDR) values for the entire set of GO processes; the number of DEGs enriching to each GO process each day is listed within each box; blank boxes represent no statistical significance. (**c**) Bar graph depicting statistically significant GO processes to which downregulated genes 6 DPI enriched; the line graph represents -log_10_(FDR) of the enriched term. (**d**) 4-way venn diagram shows overlap of DEGs (protein-coding human homologs) detected 3, 4, 5 and 6 DPI. (**e**) Heatmap representing gene expression (shown as absolute normalized RPKM values) of the common 30 DEGs first detected 3DPI and upregulated throughout infection; range of colors is based on scaled and centered RPKM values of the entire set of genes (red represents increased expression while blue represents decreased expression); each column represents the median RPKM values for each DPI.

We next determined longitudinal gene expression profiles in WB using RNA sequencing (RNA-Seq). The number of differentially expressed genes (DEGs) correlated very tightly with viral replication with large changes observed 5 and 6 DPI (Fig. 3a). The majority of DEGs mapped to human homologues (60–90%) with some DEGs remaining uncharacterized (10–40%) and a few mapping to non-coding RNA (ncRNA; 0.2–0.5%) (Supplementary Table S2). We also determined the number of ZEBOV transcripts mapping to each ZEBOV open reading frame (ORF) and intergenic region (IGR) (Supplementary Fig. S4a). We detected a significant increase in transcripts from all ORFs and IGRs 5-6 DPI. Furthermore, NP had the highest number of transcripts while VP24 and L had the lowest, indicative of a productive ZEBOV transcriptional gradient. To understand the biological impact of the gene expression changes detected each DPI, we performed functional enrichment using MetaCore^TM^ ^13^ to identify gene ontology (GO) processes, which are defined terms representing the biological processes of a gene set. DEGs upregulated 3 to 6 DPI enriched to GO processes associated with host defense (Fig. 3b). As infection progressed, both the number of DEGs mapping to these GO processes and the false discovery rate (FDR)-corrected p-values became more significant (Fig. 3b). Additionally, DEGs upregulated 5 and 6 DPI, when clinical signs are detected (Fig. 1a,b), enriched to metabolism and leukocyte activation GO processes (Fig. 3b). A significant number of downregulated DEGs was only detected 6 DPI and most of those genes enriched to GO processes associated with T-cell activation and metabolic processes (Fig. 3c).

**Figure 4:**
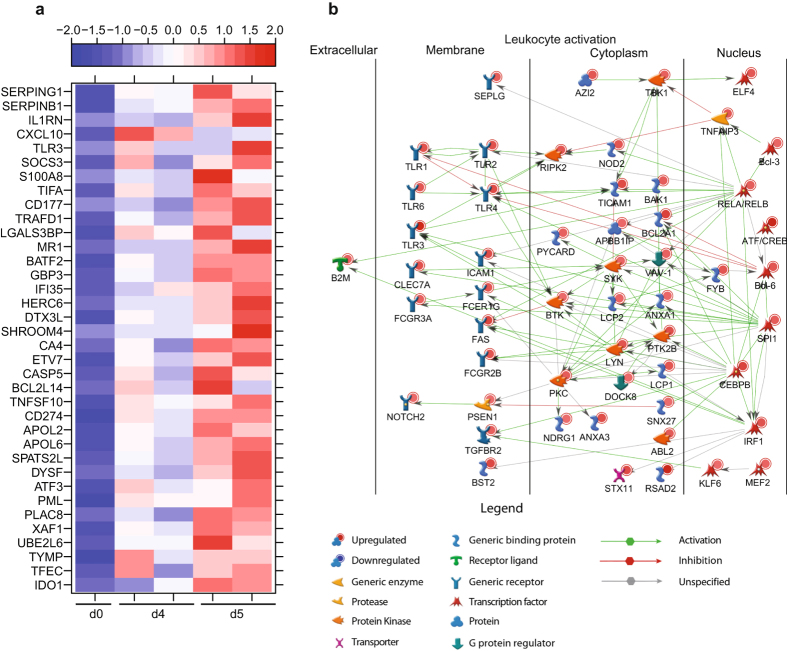
Gene expression changes 5 DPI reflect increased number of granulocytes. (**a**) Heatmap representing gene expression (shown as absolute normalized RPKM values) of DEGs upregulated 4 and 5 DPI with a FC ≥ 6.5; range of colors is based on scaled and centered RPKM values of the entire set of genes (red represents increased expression while blue represents decreased expression); day 0 is represented by the median RPKM value, while each column represents 1 animal for 4 and 5 DPI. (**b**) Network depicting direct interactions of DEGs upregulated 5 DPI that map to GO process “Leukocyte Activation”.

The 30 DEGs that were detected 3 DPI remained upregulated throughout infection with fold changes that increased from ~20 at 3 DPI to ~150 at 6 DPI (Fig. 3d). These 30 genes play a role in antiviral defense including: interferon-stimulated genes such as OAS1 (2′-5′-Oligoadenylate Synthetase 1; fold change (FC) = 31.2), *MX1* (MX Dynamin-Like GTPase 1; FC = 18.2), *IFI44* (Interferon-Induced Protein 44; FC = 16.6); and sensors of viral nucleic acids such as *DHX58* (DEXH Box Polypeptide 58; FC = 7.7) and *IFIH1* (Interferon-Induced with Helicase C Domain 1; FC = 5.3) (Fig. 3e). Similarly, the 68 upregulated genes that were newly detected 4 DPI remained upregulated 5-6 DPI (Fig. 3d). Many of these genes were involved in inflammation including *SERPING1* (Serpin Family G Member 1; FC = 202.8), *CXCL10* (FC = 38.0), *S100A8* (S100 Calcium Binding Protein A8; FC = 6.5), and *TIFA* (TRAF Interacting Protein with Forkhead Associated Domain; FC = 33.8) (Fig. 4a). Other upregulated genes in this group activate innate immunity such as *TLR3* (Toll-Like Receptor 3; FC = 34.6) and *CD177* (FC = 9.4), while others encode negative regulators e.g. *IL1RN* (Interleukin 1 Receptor Antagonist; FC = 41.3), *SOCS3* (Suppressor of Cytokine Signaling 3; FC = 23.9) and *TRAFD1* (TRAF-Type Zinc Finger Domain Containing 1; FC = 6.9). Finally, some of these genes play a role in cell death such as *TNFSF10* (FC = 11.0), *CASP5* (caspase 5; FC = 46.6), and *CD274* (Programmed Death Ligand 1; FC = 12.5) (Fig. 4a).

### End-stage disease is characterized by upregulation of genes important for cell death and inflammation and downregulation of those important for T-cell activation and translation

A substantial number of DEGs was detected 5 DPI (801). These DEGs enriched to GO processes associated with host defense as well as apoptosis and metabolism (Fig. 3b). A network analysis of the DEGs that enriched to GO processes “Leukocyte activation” showed that several were regulated by a number of transcription factors critical to the inflammatory response, notably components of the NFκB complex *RELA/RELB* (V-Rel Avian Reticuloendotheliosis Viral Oncogene Homolog A and B; FC = 5.0 and FC = 5.9) and *SPI1* (SPI-1 Proto-Oncogene; FC = 6.1) (Fig. 4b). Interestingly, genes involved in B-cell development such as *BCL*6 (B-Cell CLL/Lymphoma 6; FC = 8.3) and *BTK* (Bruton Agammaglobulinemia Tyrosine Kinase; FC = 3.4) as well as T-cell activation including *LCP* (Lymphocyte Cytosolic Protein 1; FC = 7.4) and *FYB* (FYN Binding Protein; FC = 8.2) were upregulated. Moreover, Toll-Like Receptors *(TLR) 1-4* and *6* were upregulated (FC = 9.5, 10.1, 102.9, 12.1, and 8.2, respectively) (Fig. 4b).

**Figure 5:**
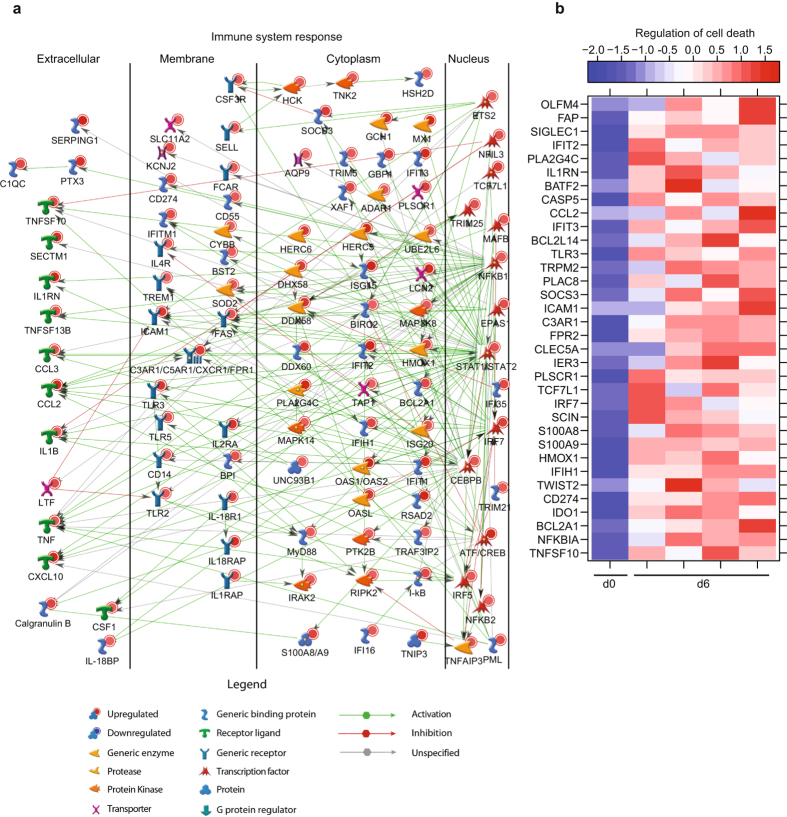
End-stage disease is characterized by upregulation of genes important for cell death and inflammation. (**a**) Network depicting direct interactions of DEGs upregulated 6 DPI that map to “Immune system response” with a FC ≥ 5.6. (**b**) Heatmap representing gene expression (shown as absolute normalized RPKM values) of DEGs upregulated 6 DPI that map to “Regulation of cell death” with a FC ≥ 16; range of colors is based on scaled and centered RPKM values of the entire set of genes (red represents increased expression while blue represents decreased expression); day 0 is represented by the median RPKM value, while each column represents 1 animal for 6 DPI.

Similarly, DEGs upregulated 6 DPI enriched to GO processes associated with host defense, cell death, and metabolic processes (Fig. 3b). Of the 409 genes that mapped to “Immune system process”, 110 with known roles in inflammation directly interacted with one another (Fig. 5a). Expression of these genes is regulated by: *NFKB1* (Nuclear Factor of Kappa Light Polypeptide Gene Enhancer in B-Cells 1; FC = 3.0); *STAT1/STAT2* (Signal Transducer and Activator of Transcription 1 and 2; FC = 7.3 and FC = 12.5); and *CEBPB* (CCAAT/Enhancer Binding Protein, Beta; FC = 10.0). DEGs regulated by these transcription factors included chemokines/cytokines such as *CCL2* (FC = 123.7), *CXCL10* (FC = 217.3), *TNF* (Tumor Necrosis Factor; FC = 8.3), and *IL1B* (FC = 7.2); as well as their receptors, notably *C3AR1* (Complement Component 3a Receptor 1; FC = 39.7), *CXCR1* (FC = 4.7), and *IL1RAP* (Interleukin 1 Receptor Accessory Protein; FC = 19.9). Several IFN-stimulated genes (*ISG15*, *IFIT2*) and RNA helicases (*HERC5* and *DHX58*) were also part of this network. Other DEGs in this network play a role in generating or clearing reactive oxygen species e.g. *CYBB* (Cytochrome B-245, Beta; FC = 5.9) and *SOD2* (Superoxide Dismutase 2; FC = 9.6) (Fig. 5a). These upregulated genes are in line with the dysregulated immune activation often attributed to Ebola hemorrhagic fever (EHF). DEGs upregulated 6 DPI also play a role in 1) cell death including *FAS* (Fas Cell Surface Death Receptor; FC = 11.2), *TNFSF10* (FC = 16.6), *BCL2A1* (BCL2 Related Protein A1; FC = 18.7), *TRPM2* (Transient Receptor Potential Cation Channel Subfamily M Member 2; FC = 47.9); or 2) cell cycle progression such as *TCF7L1* (Transcription Factor 7 Like 1; FC = 32.1) and *OLFM4* (Olfactomedin 4; FC = 981.8) (Fig. 5a,b).

**Figure 6:**
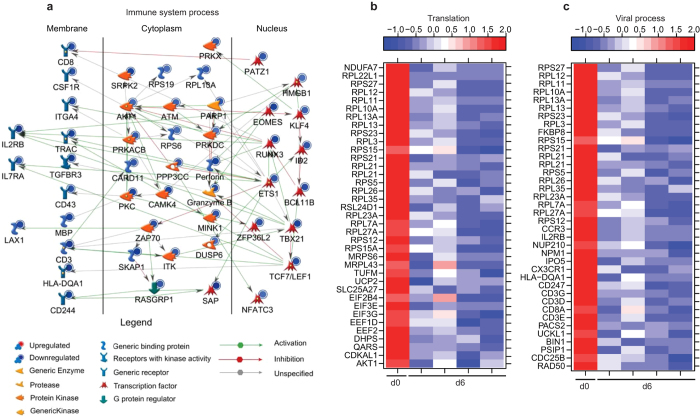
ZEBOV-Makona pathogenesis is characterized by downregulation of genes critical for T-cell activation and translation. (**a**) Network depicting direct interactions of DEGs downregulated 6 DPI that map to “Immune system response”. (**b**,**c**) Heatmap representing gene expression (shown as absolute normalized RPKM values) of DEGs downregulated 6 DPI that map to “Translation” with a FC ≥ 2.5 (**b**) and DEGs downregulated 6 DPI that map to “Viral process” with a FC ≥ 3.0 (**c**); range of colors is based on scaled and centered RPKM values of the entire set of genes (red represents increased expression while blue represents decreased expression); day 0 is represented by the median RPKM value, while each column represents 1 animal for 6 DPI.

At day 6, changes in gene expression were indicative of a suppressed adaptive immune response and cellular homeostasis (Fig. 3c). Specifically, several of the 48 downregulated genes mapping to “Immune system process” play a role in T-cell responses: *CD3* (FC = 6.8), *CD8* (FC = 4.8), *IL2RB* (FC = 9.3), *TRAC* (T-Cell Receptor Alpha Constant; FC = 6.7), and *ZAP70* (Zeta-Chain (TCR) Associated Protein Kinase; FC = 4.9) (Fig. 6a). Additionally, genes encoding effector molecules *GZMB* (Granzyme B; FC = 5.2), *PRF1* (Perforin; FC = 7.1), and *CD244* (Natural Killer Cell Receptor 2B4; FC = 7.3) were downregulated (Fig. 6a). The second major group of downregulated genes enriched to GO process “translation” (Fig. 3c) and contained translation initiation factors e.g. *EIF3E* (Eukaryotic translation initiation factor 3 subunit E; FC = 2.6); elongation factors such as *EEF2* (Elongation Factor 2; FC = 2.5); and ribosomal proteins e.g. *RPL22L1* (Ribosomal protein L22 Like 1; FC = 12.9) (Fig. 6b). Interestingly, several of the genes that enriched to the GO term “viral process” were also ribosomal proteins e.g. *RPS27* (FC = 9.6) as well as genes that regulate nuclear import such as importins (e.g., *IPO5*; FC = 2.6) and nucleoporins (e.g., *NUP210*; FC = 2.8) (Fig. 6c).

### DEGs detected only in PBMC fraction play a role in regulating blood clotting, response to oxidative stress and vasculature development

**Figure 7:**
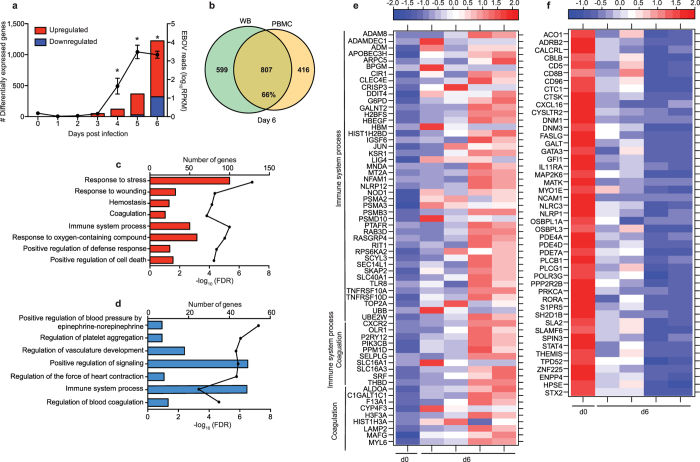
DEGs detected only in PBMC fraction play a role in regulating blood clotting, response to oxidative stress, and vasculature development (**a**) Bar graph depicts number of protein-coding differentially expressed genes (DEGs; defined as those ≥log2 fold change compared to 0 DPI and FDR-corrected p-value ≤ 0.05) that have human homologues. Line graph indicates number of viral transcripts reported as normalized by reads per kilobase per million mapped (RPKM); the *EdgeR* package was used to determine statistically significant changes in viral reads; *denote p-value ≤ 0.05 at the indicated time point compared to 0 DPI. (**b**) Venn diagram shows overlap between DEGs in PBMC and WB 6 DPI. (**c**,**d**) Bar graph depicting statistically significant GO processes to which upregulated (**c**) and downregulated (**d**) genes found exclusively in PBMC 6 DPI enriched; the line graph represents −log_10_(FDR) of the enriched term. (**e**,**f**) Heatmap representing gene expression (shown as absolute normalized RPKM values) of DEGs upregulated 6 DPI that map to “Coagulation” and/or “Immune system process” (**e**) and DEGs downregulated 6 DPI that map to “Immune system process” (**f**); range of colors is based on scaled and centered RPKM values of the entire set of genes (red represents increased expression while blue represents decreased expression); day 0 is represented by the median RPKM value, while each column represents 1 animal for 6 DPI.

In order to get a better understanding of the contributions of lymphocytes and antigen-presenting cells to gene expression changes, we next identified transcriptional changes within PBMC, which are devoid of granulocytes, erythrocytes, and platelets. The kinetics of host and viral gene expression changes in PBMC were similar as those described for WB, but the number of DEGs and viral reads was significantly smaller (Fig. 7a and Supplementary Fig. S4b). As described for WB, 45 mostly interferon-stimulated genes were upregulated 3-6 DPI in PBMC with fold changes that increased from ~10 at 3 DPI to ~70 at 6 DPI as infection progressed (Supplementary Fig. S5a,b). Of these 45 DEGs, 27 were also detected in WB 3 DPI (Supplementary Fig. S5b). The remaining 18 DEGs were detected in WB 4-5 DPI. Overall, a substantial number (50–75%) of DEGs detected in PBMC throughout infection were also detected in WB (Supplementary Fig. S5c–e and Fig. 7b).

Most of the 59 DEGs detected only in PBMC 4 DPI, were detected in WB 5-6 DPI such as *C1QC* (Complement Component 1, Q Subcomponent, C Chain; FC = 6.6), *BCL2A1* (FC = 3.8), and *CYBB* (FC = 3.1) (Supplementary Fig. S5f). DEGs detected only in PBMC 5 DPI enriched to GO processes associated with blood regulation and response to oxidative stress (Supplementary Fig. S5g) including *ADAMDEC1* (A Disintegrin and Metalloproteinase Domain-Like Protein Decysin-1; FC = 20.2), *NCF1* (Neutrophil Cytosolic Factor 1; FC = 3.1), *ATP2A2* (ATPase Sarcoplasmic/Endoplasmic Reticulum Ca2+ Transporting 2; FC = 2.5), *THBS1* (Thrombospondin 1; FC = 3.3), and *F13A1* (Coagulation Factor XIII A Chain; FC = 4.4) (Supplementary Fig. S5h).

Differences between the PBMC and WB gene expression profiles were most prominent 6 DPI (Fig. 7b–d). DEGs upregulated only in PBMC enriched to immune related GO processes such as host defense and coagulation (Fig. 7c). Upregulated genes that enriched to “Immune system process” include *MNDA* (Myeloid Cell Nuclear Differentiation Antigen; FC = 2.6) and *NLRP12* (PYRIN-Containing APAF1-Like Protein 7; FC = 8.9). Genes that play a role in coagulation include *PTAFR* (Platelet Activating Factor Receptor; FC = 6.7) and *THBD* (Thrombomodulin; FC = 15.8) (Fig. 7e). Some of the downregulated genes detected only in PBMC 6 DPI play a role in host defense such as: *CD96* (FC = 3.5), *CD8B* (FC = 2.6), and *SLAMF6* (Activating NK Receptor; FC = 2.1) (Fig. 7d,f). Others were involved in vasculature development such as *CYSLTR2* (Cysteinyl Leukotriene Receptor 2; FC = 3.4) and *ADRB2* (Beta-2 Adrenoreceptor; FC = 3.3), as well as coagulation e.g. *PRKCA* (Protein Kinase C, Alpha; FC = 2.9) and *ENPP4* (Ectonucleotide Pyrophosphatase 4; FC = 2.4) (Fig. 7f). To better understand the source of the DEGs detected only in PBMC 6 DPI, we used the Immunological Genome Project Consortium database (ImmGen), a collaborative effort to delineate gene expression patterns across different leukocyte subsets^14^ (Supplementary Fig. S6). This analysis revealed that most of these DEGs are expressed by DCs and monocytes/macrophages.

### ZEBOV-Makona induces overlapping but distinct gene expression changes compared to ZEBOV-Kikwit

To better understand the differences in pathogenesis caused by ZEBOV-Makona compared to the previously identified ZEBOV variant Kikwit, we compared our transcriptome results to those obtained from a recent study in which male cynomolgus macaques were challenged intramuscularly with 1000 PFU of Kikwit^15, 16^. Protocols for library preparation, sequencing, and bioinformatics analysis were the same, making these comparisons feasible. This comparison further supports delayed disease progression following challenge with Makona relative to Kikwit (Supplementary Fig. S7). Specifically, we observed significantly larger gene expression changes in both WB and PBMC 4 DPI following Kikwit infection compared to Makona (Supplementary Fig. S7a,b). Functional enrichment showed that DEGs detected 4 days following both Kikwit and Makona infection enriched to innate immunity while lymphocyte related transcripts were only downregulated following Kikwit infection, indicating lymphopenia occurs earlier compared to Makona infection^17–19^ (Supplementary Fig. S7c,d).

**Figure 8:**
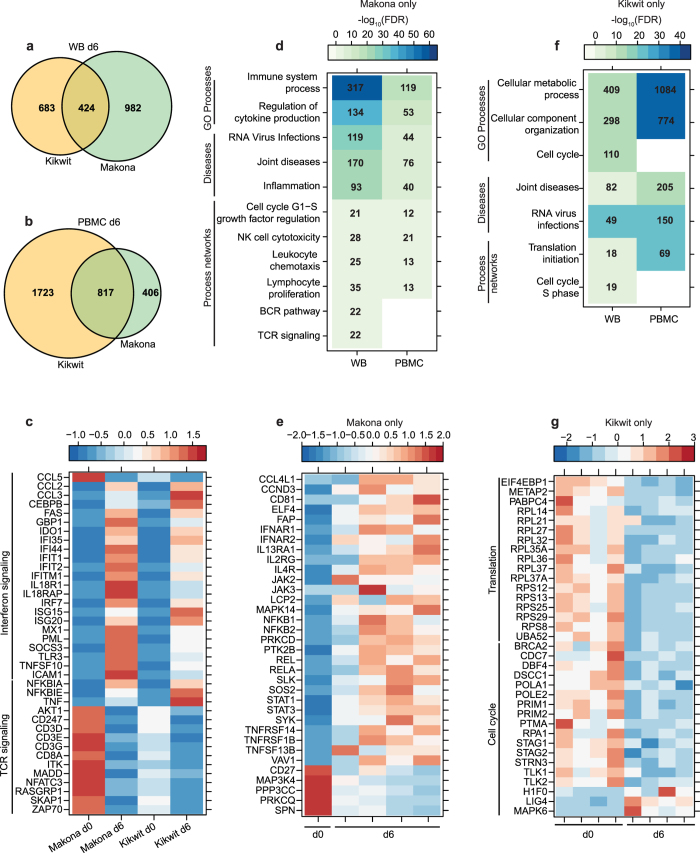
Comparison of host transcriptional profile following ZEBOV-Makona or ZEBOV-Kikwit infection at 6DPI. (**a**,**b**) Venn diagram shows overlap between DEGs detected following Kikwit and Makona infection 6 DPI in WB (**a**) and PBMC (**b**). (**c**) Heatmap representing gene expression (shown as absolute normalized RPKM values) of shared DEGs detected 6 DPI following Kikwit or Makona infection that enriched to Process Network terms “Inflammation – Interferon signaling” and “Immune response – TCR signaling”; range of colors is based on scaled and centered RPKM values of the entire set of genes (red represents increased expression while blue represents decreased expression); each column represents the median RPKM values on each day for either Kikwit or Makona infection. (**d**) Heatmap representing functional enrichment of DEGs exclusively detected following Makona infection 6 DPI; color intensity represents the statistical significance (shown as -log_10_ of the FDR-corrected p-Value); range of colors is based on the lowest and highest −log_10_(FDR) values for the entire set of terms; the number of DEGs enriching to each functional enrichment term each day is listed within each box; blank boxes represent no statistical significance. (**e**) Heatmap representing gene expression (shown as absolute normalized RPKM values) of DEGs found only 6 DPI with Makona that enriched to “Lymphocyte proliferation”; day 0 is represented by the median RPKM value, while each column represents 1 animal for 6 DPI. (**f**) Heatmap representing functional enrichment of DEGs exclusively detected following Kikwit infection 6 DPI. (**g**) Heatmap representing gene expression (shown as absolute normalized RPKM values) of DEGs found exclusively 6 DPI following Kikwit infection that enriched to “Translation- initiation” and “Cell cycle - S phase”; each column represents 1 animal 6 DPI.

By 6 DPI, widespread transcriptional responses were detected following infection with either variant and although there was significant overlap, we identified distinct gene expression profiles unique to Kikwit and Makona infection (Fig. 8a,b). By 6 DPI, DEGs associated with inflammation and lymphopenia were detected with either variant. Since whole blood encompasses PBMC and functional enrichment was similar for both, we focused our analysis on differences between Kikwit and Makona infection in WB 6 DPI. Infection with either strain increased expression of ISGs (*GBP1*, *IFIT1*, *IRF7*) and reduced expression of lymphocyte related genes (*CD3D*, *CD8A*, *ZAP70*) (Fig. 8c). However, DEGs unique to Makona infection suggest a more robust immune dysregulation (Fig. 8d,e), while DEGs found exclusively during Kikwit infection were mostly involved with dysregulation of metabolism, cell cycle, and translation (Fig. 8f,g).

## Discussion

Although ZEBOV-Makona has high sequence homology to ZEBOV variants Mayinga and Kikwit, data from a recent study suggest Makona infection progresses less rapidly^12, 17^. However, the biological processes underlying altered disease course by ZEBOV-Makona is currently understudied. Therefore, we integrated flow cytometry and RNA-sequencing with clinical data to gain a more comprehensive understanding of ZEBOV-Makona pathogenesis. Here, we report that macaques infected with ZEBOV-Makona (isolate C07) presented with clinical signs typical of EHF including anorexia, hunching, fever, rash, and hemorrhaging. Clinical chemistry was also consistent with EHF and indicated damage to multiple organs, notably kidney, pancreas and liver. Moreover, lymphopenia and thrombocytopenia were observed at the later stages of disease together with sharp increases in plasma levels of proinflammatory cytokines (IL-1β, IL-18, IL-6, and IFNα) and chemokines (I-TAC, MCP-1, and eotaxin), also characteristic of EHF.

Clinical scores, changes in liver and kidney enzymes, cytokine and chemokine levels, and pathology scores appeared later and were less severe than those previously reported in macaques infected with either the ZEBOV variants Mayinga or Kikwit^6, 12^, despite comparable levels of viremia. It should be noted that early Makona isolates, including the C07 isolate used in this study, lack the A82V mutation in GP, which was observed in later Makona isolates and shown to confer increased infectivity of primate cells^20, 21^. Comparison of transcriptional changes also indicated that infection with ZEBOV-Makona established a distinct host transcriptional profile compared to the ZEBOV-Kikwit variant, characterized by more robust changes in genes important for immunity and less pronounced changes in cellular metabolism and apoptotic pathways. It is possible that the delayed progression of disease led to enhanced immune activation following ZEBOV-Makona infection.

As previously reported for Mayinga and Kikwit^17, 22–24^, ZEBOV-Makona infection induced early and sustained upregulation of genes important for antiviral immunity, notably ISGs. However, this increase was detected a day later than what was reported for ZEBOV-Kikwit infected macaques^17^. The initial upregulation of ISGs in our study correlated with the increased frequency of pDCs, which are potent producers of type I IFN. The magnitude of ISG expression was highest 6 DPI and correlated with levels of circulating IFNα, which at the later stages of disease may be secreted from either infected APCs or non-infected cells that are activated by viral debris. Additionally, the increased transcription of innate immune genes 4 DPI correlated with increased frequency of mDC and the upregulation of activation marker CD80 on all DC subsets.

These data contrast *in vitro* studies that show ZEBOV VP35 and VP24 prevent production and cellular responses to type I IFN, including failure to up-regulate CD80 and CD86 and mature into mobile antigen-presenting cells^25–27^. However, this discrepancy could be due to the infection rate likely being lower *in vivo*. Non-infected DCs may become activated by “shed” GP, which was recently shown to induce the secretion of pro-inflammatory cytokines and expression of co-stimulatory molecules CD40, CD80, CD83, and CD86 on monocyte-derived DCs^28^. These data highlight the importance of delineating transcriptional profiles of individual immune cell subsets following *in vivo* infection in order to determine precisely how and in which cells ZEBOV evade host innate, and consequently, adaptive immune responses.

The sustained Type I IFN response may lead to suppression of the adaptive immune response as recently reported in a mouse model of chronic lymphocytic choriomeningitis virus (LCMV) infection, where production of the immunosuppressive mediators IL-10 and PD-L1 was dependent on Type I IFN signaling^29, 30^. Indeed, following upregulation of ISGs, we observed increased transcription of PD-L1, which preceded lymphopenia and reduced expression of genes typically expressed by lymphocytes. Therefore, it is possible that the high levels of circulating type I IFN contribute to the suppression of the adaptive immune responses following ZEBOV infection. The increased expression of PD-L1 is in line with recent clinical studies that reported increased numbers of CD4^+^ and CD8^+^ T-cells expressing the inhibitory molecules CTLA-4 and PD-1 in ZEBOV-Makona fatalities^31^.

Although the number of monocytes was unchanged, the percentage of non-classical monocytes (CD16^+^) significantly increased 4 DPI. Non-classical monocytes can produce large amounts of pro-inflammatory cytokines in an acute inflammatory state^32, 33^ and may be largely responsible for the upregulation of proinflammatory mediators (*SERPING1*, *CXCL10*, *S100A8*, *TIFA*) 4 DPI. “Shed” GP has also been shown to bind and activate monocytes, resulting in the production of inflammatory cytokines^28^. Our data contrasts a recent study reporting decreased levels of non-classical CD16^+^ monocytes in patients diagnosed with EHF in Guinea compared to febrile patients who tested negative for EHF^34^. However, the increase we observed was transient and preceded clinical signs. It is possible that the difficulties associated with a longitudinal study in a clinical setting precluded detection of early monocyte activation. Interestingly, we observed a transient increase in cytokine-producing NK cells 1 DPI. However, its clinical significance is unclear.

As infection progressed, we detected a robust upregulation of genes encoding cytokines and chemokines (*CCL2*, *CCL3*, *IL1B*, *CXCR1*, *IL1RN*) as well as granulocyte associated transcripts (*TLRs 1-4*, *SPI1*, *S100A8*, *S100A9*, *CD177*), which correlated with the higher numbers of granulocytes 5 DPI. Granulocytosis was also reported following ZEBOV-Kikwit infection, albeit earlier at 4 DPI^6^. It is possible that the acute viral infection as well as the proinflammatory environment induced hematopoietic differentiation within the bone marrow, resulting in increased numbers of neutrophils^35, 36^. The largest gene expression changes (1400) were identified 6 DPI when a substantial decrease in many lymphocyte-related transcripts was detected (*CD3, CD8, IL2RB, LAX1*, and *ZAP70, GZMB, PRF1*). These changes correlated with a drop in CD4 T-cells, CD8 T-cells, B-cells, and NK-cells. Interestingly, proliferation of CD4 and CD8 T-cells increased 4-6 DPI, possibly as an attempt to restore their numbers. We also detected a downregulation of genes associated with translation and viral process 6 DPI such as translation initiation factors, elongation factors, and ribosomal proteins. These decreased transcripts suggest either cell death and/or an attempt by the host defense to prevent ZEBOV replication.

These observations are similar to the recent study by Liu and Speranza *et al*.^37^, in which 2200 DEGs were identified in the WB from 88 patients who succumbed to ZEBOV-Makona infection (average of 5.9 days until symptom onset). These 2200 DEGs enriched to comparable biological processes reported in this study, notably those associated with innate immunity, Type I interferon signaling, response to cytokine, cell death, T-cell activation, and coagulation. Moreover, in line with the early and sustained detection of ISGs in our study, Liu and Speranza *et al*. reported a higher number of ISGs in fatalities compared to surviving patients.

This study is the first to report a simultaneous analysis of gene expression changes within WB and PBMC from the same animals following ZEBOV infection. Interestingly, DEGs detected only in PBMC enriched to GO processes “Blood coagulation”, “Response to oxidative stress”, and “Vasculature development” 5-6 DPI. Further bioinformatics analysis confirmed that the majority of the DEGs unique in PBMC 5-6 DPI are primarily expressed by monocytes/macrophages and DCs. Upregulation of genes that enrich to response to oxidative stress may be attributed to reactive oxygen species produced by monocytes as suggested by increased transcripts of *NCF1*, an oxidase that can produce superoxide anion, and *CYBB*, a component of the microbicidal oxidase system of phagocytes^38^. Moreover, upregulation of DEGs that play a role in coagulation in PBMC including *PTAFR*, *THBD*, *P2RY12*, and *PRKCA* is in line with the potential role of ZEBOV-infected monocytes in initiating intra-vascular coagulation. Future studies will focus on transcriptional changes within ZEBOV-infected monocytes to assess to what degree inflammation, production of reactive oxygen species, and initiation of coagulation can be attributed to infected monocytes. Moreover, DEGs exclusively detected in WB suggest a significant role for granulocytes and platelets in ZEBOV-Makona pathogenesis.

## Materials and Methods

### Virus and Challenge

A laboratory seed stock of the Makona variant was grown from the serum of a 2014 fatal human case in Guékédou, Guinea and passaged twice in authenticated Vero E6 cells (ATCC, CRL-1586) to produce ZEBOV isolate H.sapiens-tc/GIN/2014/Makona-Gueckedou-C07, accession number KJ660347.2. This clone is one of the earliest isolates from the recent EBOV epidemic and was used in recent nonhuman primate studies^12^. The University of Texas Medical Branch at Galveston Institutional Animal Care and Use Committee (IACUC) and the Institutional Biosafety Committee approved this study and all protocols in accordance with state and federal statutes and regulations relating to experiments involving animals. Ten healthy, filovirus-negative male cynomolgus macaques 3–5 years of age and between 4–8 kg were challenged with 1000 pfu of ZEBOV-Makona intramuscularly with the dose divided equally into the left and right quadriceps. Animals were housed in the Biosafety Level 4 (BSL-4) laboratory in the Galveston National Laboratory (GNL) and monitored post challenge for clinical signs of disease.

### Sample Collection and PBMC isolation

Blood was collected by venipuncture into EDTA and serum tubes according to the study design (Fig. 1a). WB was added to AVL buffer (Qiagen, Valencia, CA) to isolate RNA. To separate plasma and serum, tubes were spun at 2500 rpm for 10 minutes at 4 °C. EDTA plasma and serum were stored at −80 °C for future analysis and virus quantification. To isolate PBMC, WB was centrifuged over Histopaque (Sigma-Aldrich, St. Louis, MO) using AccuSpin Tubes (Sigma-Aldrich, St. Louis, MO) at 1400 rpm for 45 minutes, room temperature with no brake. The PBMC buffy coat was extracted and washed in RPMI media. Isolated cells were counted on a TC20 Automated Cell Counter (Bio-Rad, Hercules, CA). 1.0 × 10^6^ PBMC were put into Trizol (Invivogen) buffer for RNA isolation. An additional 5.0 × 10^6^ PBMC were used for flow cytometry analysis. Remaining PBMC cells were frozen and stored at −80 °C (stable for 6 months) for future analysis.

### Hematology and Clinical Chemistry

WB collected in EDTA was analyzed by a hematological analyzer (Beckman Coulter, Brea, CA) that measures: white blood cell and differentials, red blood cells, platelets, hematocrit values, hemoglobin, mean cell volumes, mean corpuscular volumes, and mean corpuscular hemoglobin concentrations. Serum was collected for clinical chemistry analysis using a Piccolo point-of-care analyzer and Biochemistry Panel Plus analyzer discs (Abaxis, Sunnyvale, CA) to measure concentrations of albumin, amylase, ALT, AST, ALP, GGT, glucose, cholesterol, total protein, total bilirubin (TBIL), blood urea nitrogen (BUN), CRE, and CRP.

### Virus detection and quantification

Virus titer was measured by plaque assay on Vero E6 cells. Cells were plated in 6-well plates and grown to confluency. Virus was titrated from 10^-1^ to 10^-6^ in duplicate. Plaques were counted using neutral red stain; limit of detection was 25 PFU/ml.

RNA was isolated from WB using AVL Buffer and Viral RNA mini-kit (Qiagen, Valencia, CA). As previously described, primers/probe targeting the VP30 gene of ZEBOV-Makona were used for RT-qPCR^39^. EBOV RNA was detected using the CFX96 detection system (BioRad Laboratories) using One-Step Probe qRT-PCR Kits (Qiagen) with the following cycle conditions: 50 °C for 10 minutes, 95 °C for 10 seconds, and 40 cycles of 95 °C for 10 seconds followed by 59 °C for 30 seconds. Threshold cycle values representing ZEBOV genome equivalents (GEq) were analyzed with CFX Manager Software, and data are shown as means ± SD of technical replicates. To create the GEq standard, RNA from ZEBOV stocks was extracted and the number of ZEBOV genomes calculated using Avogadro’s number and the molecular weight of the ZEBOV genome.

### Cytokine, Chemokine and Growth Factor Analysis

Circulating cytokines were measured in the plasma using NHP Cytokine/Chemokine/Growth factor (eBioscience, San Diego CA) 37-plex panel that measures IFNγ, IFNα, TNFα, IL1RA, IL1b, IL2, IL4, IL5, IL6, IL7, IL8, IL10, IL12p70, IL13, IL15, IL17A, IL18, IP10, IL23, sCD40L, SCF, MCP1, MIP1α, MIP1β, MIG, Eotaxin, ITAC, BLC, SDF1α, VEGFA, VEGFD, GCSF, GMCSF, BDNF, FGF2, NGFß, and PDGFBB.

### Flow Cytometry Analysis

PBMC were stained in round bottom 96-well plates using 5 different panels to measure frequency and characterize phenotype of monocytes and dendritic cells (CD3, CD20, CD14, HLA-DR, CD16, CD11c, CD123), T-cells (CD4, CD8b, CD28, CD95, CCR7, Ki67), B-cells (CD20, IgD, CD27, Ki67), dendritic cell activation (CD3, CD20, CD14, HLA-DR, CD16, CD11c, CD123, CD80), and natural killer cells (CD3, CD20, CD8a, CD159a, Granzyme B, and CD16)^40^. Antibodies used are detailed in Supplementary Table S3. For all panels, cells were stained and fixed according to manufacturer recommendations (Tonbo Biosciences, San Diego, CA). For panels requiring intracellular stains, cells were stained, fixed and permeabilized using CytoFix/CytoPerm (BD Biosciences, San Jose, CA), according to manufacturer recommendations. All samples were acquired using BD FACS Diva software on a BD FACS Canto-II Flow Cytometer (Becton Dickinson Biosciences, San Jose, CA). Live cells were identified by FSC and SSC and a minimum of 50,000 events were collected for each sample. Data were analyzed using FlowJo Analysis Software (FlowJo LLC, Ashland, OR) and Prism Software (Irvine, CA). Gating strategy is shown in Supplementary Fig. S2.

### Library preparation for RNA Seq

RNA from WB and PBMC was isolated using Zymo Research Direct-zol RNA mini-prep (Zymo Research) per manufacturer’s instructions. Concentration and integrity of RNA was determined using an Agilent 2100 Bioanalyzer. Ribosomal RNA (rRNA) was depleted using the ClontechRibo-Gone rRNA Removal kit. Libraries were constructed using the ClontechSMARTer Stranded RNA-Seq kit. First, rRNA-depleted RNA was fragmented and converted cDNA. Adapters were ligated and the ~300 base pair (bp) fragments were then amplified by PCR and selected by size exclusion. Each library was prepared with a unique indexed primer for multiplexing. Quantitation and quality of libraries were confirmed on the Agilent 2100 Bioanalyzer. Multiplexed libraries were subjected to single-end 100 bp sequencing using the Illumina HiSeq. 2500 platform. RNA-sequencing data presented in this article were submitted to the National Center for Biotechnology Information Sequence Read Archive (Accession number PRJNA398558).

### Bioinformatic analysis

Data analysis was performed with the RNA-Seq workflow module of the systemPipeR package available on Bioconductor^41^ as previously described^16^. RNA-Seq reads were trimmed using Trim Galore with an average phred score cutoff of 30 and minimum length of 75 bp; 3 bp from the 5′ end were trimmed as per Clontech’s instruction. The *Macaca mulatta* genome sequence (Macaca_mulatta.MMUL_1.dna.toplevel.fa) and annotation file from Ensembl (Macaca_mulatta.MMUL_1.78.gtf) was used for alignment. In order to determine the kinetics of viral transcription, the ZEBOV-Makona genome (H.sapiens-wt/GIN/2014/Makona- Gueckedou-C07) from Virus Pathogen Resource was adjoined to the *Macaca mulatta* reference. ZEBOV open reading frames (ORFs), intergenic regions (IGRs) and leader and trailing sequences were defined based on the ZEBOV-Makona genome annotation GTF file: NP (470–2689), VP35 (3129–4151), VP40 (4479–5459), GP (6039–8068), VP30 (8509–9375), VP24(10345–11100), L (11581–18219), Leader (1–469), IGR_NP_VP35 (2690–3128), IGR_VP35_VP40 (4152–4478), IGR_VP40_GP (5460–6038), IGR_GP_VP30 (8069–8508), IGR_VP30_VP24 (9376–10344), IGR_VP24_L (11101–11580), Trailing (18220–18959). RNA-Seq reads were aligned to a reference genome containing *Macaca mulatta* and ZEBOV-Makona genome sequences using Bowtie2/Tophat2. Statistical analysis of differentially expressed genes (DEGs) was performed using the *edgeR* package, which normalizes reads by the trimmed mean of M values (TMM) method. DEGs were defined as those with a fold change ≥ 2 compared to 0 DPI and a false discovery rate (FDR) corrected p-value ≤ 0.05. Only protein coding genes with human homologs (Supplementary Table S2) and an average of 5 reads per kilobase of transcript per million mapped reads (RPKM) were included for further analysis. Reads mapping to the ZEBOV-Makona genome were also normalized as RPKM. Statistical analysis of changes in normalized reads mapping to ZEBOV-Makona ORF, IGR, leader, trailing sequences, and entire genome was performed using *edgeR*. Heatmaps and venn diagrams were generated using R packages gplot and VennDiagram.

### Functional enrichment

Functional enrichment of DEGs was done to identify clusters of genes mapping to specific biological pathways including Gene Ontology (GO) processes, Disease terms and Process networks using MetaCore^TM^ (Thomson Reuters, New York, NY). Since this software requires human gene identifiers for analysis, rhesus DEGs were mapped to human homologs using BioMart (Supplementary Table S2).

### Statistical analysis

Statistical analysis of viral genome copy number; hematology and clinical chemistry data; cytokine, chemokine, and growth factor data; and flow cytometry data was carried out using the SAS software, PROC MIXED. A repeated measures analysis was used to model each of the dependent variables. Intra-animal correlation was modeled using a compound symmetric variance-covariance structure. In some cases, a linear trend was used for the mean. In other cases, in which a linear trend was not a good fit, a nonparametric trend was used where each time point was modeled by its own mean. Missing data was handled by using maximum likelihood algorithms to fit the model. When a linear model was an adequate trend, the p-value for the estimated slope was reported. When each time point was modeled by its own mean, the mean response at each non-zero time point was contrasted with the mean response at the zero time point. Holm’s multiple comparison method was used to adjust the p-values for each contrast^42^.

## Electronic supplementary material

Supplementary Information
