# Author Correction: Infection with the Makona variant results in a delayed and distinct host immune response compared to previous Ebola virus variants

**DOI:** 10.1038/s41598-019-42949-6

**Published:** 2019-05-08

**Authors:** Krista Versteeg, Andrea R. Menicucci, Courtney Woolsey, Chad E. Mire, Joan B. Geisbert, Robert W. Cross, Krystle N. Agans, Daniel Jeske, Ilhem Messaoudi, Thomas W. Geisbert

**Affiliations:** 1 Galveston National Laboratory, Galveston, TX USA; 2 Department of Microbiology and Immunology, University of Texas Medical Branch, Galveston, TX USA; 3 Division of Biomedical Sciences, University of California-Riverside, Riverside, CA USA; 4 Department of Molecular Biology and Biochemistry, University of California-Irvine, Irvine, CA USA

Correction to: *Scientific Reports* 10.1038/s41598-017-09963-y, published online 29 August 2017

This Article contains errors. The gene expression changes of Kikwit infected animals were on 7 DPI, not 6 DPI as reported.

As a result, in the Results section under the subheading ‘ZEBOV-Makona induces overlapping but distinct gene expression changes compared to ZEBOV-Kikwit’,

“By 6 DPI, widespread transcriptional responses were detected following infection with either variant and although there was significant overlap, we identified distinct gene expression profiles unique to Kikwit and Makona infection (Fig. 8a,b). By 6 DPI, DEGs associated with inflammation and lymphopenia were detected with either variant. Since whole blood encompasses PBMC and functional enrichment was similar for both, we focused our analysis on differences between Kikwit and Makona infection in WB 6 DPI.”

should read:

“At the terminal time point, widespread transcriptional responses were detected following infection with either variant and although there was significant overlap, we identified distinct gene expression profiles unique to Kikwit and Makona infection (Fig. 8a,b). DEGs associated with inflammation and lymphopenia were detected with either variant. Since whole blood encompasses PBMC and functional enrichment was similar for both, we focused our analysis on differences between Kikwit and Makona infection in WB at the end stage of disease.”

In addition, in the legend of Figure 8,

“Comparison of host transcriptional profile following ZEBOV-Makona or ZEBOV-Kikwit infection at 6DPI. (**a**,**b**) Venn diagram shows overlap between DEGs detected following Kikwit and Makona infection 6 DPI in WB (**a**) and PBMC (**b**). (**c**) Heatmap representing gene expression (shown as absolute normalized RPKM values) of shared DEGs detected 6 DPI following Kikwit or Makona infection that enriched to Process Network terms “Inflammation – Interferon signaling” and “Immune response – TCR signaling”; range of colors is based on scaled and centered RPKM values of the entire set of genes (red represents increased expression while blue represents decreased expression); each column represents the median RPKM values on each day for either Kikwit or Makona infection.”

should read:

“Comparison of host transcriptional profile following ZEBOV-Makona or ZEBOV-Kikwit infection at end stage of disease. (**a**,**b**) Venn diagram shows overlap between DEGs detected following Makona and Kikwit infection 6 and 7 DPI, respectively, in WB **(a)** and PBMC (**b**). (**c**) Heatmap representing gene expression (shown as absolute normalized RPKM values) of shared DEGs detected at end stage of disease following Kikwit or Makona infection that enriched to Process Network terms “Inflammation – Interferon signaling” and “Immune response – TCR signaling”; range of colors is based on scaled and centered RPKM values of the entire set of genes (red represents increased expression while blue represents decreased expression); each column represents the median RPKM values on each day for either Kikwit or Makona infection.”

and,

“(**f**) Heatmap representing functional enrichment of DEGs exclusively detected following Kikwit infection 6 DPI. (**g**) Heatmap representing gene expression (shown as absolute normalized RPKM values) of DEGs found exclusively 6 DPI following Kikwit infection that enriched to “Translation- initiation” and “Cell cycle - S phase”; each column represents 1 animal 6 DPI.”

should read:

“(**f**) Heatmap representing functional enrichment of DEGs exclusively detected following Kikwit infection 7 DPI. (**g**) Heatmap representing gene expression (shown as absolute normalized RPKM values) of DEGs found exclusively 7 DPI following Kikwit infection that enriched to “Translation” and “Cell cycle”; each column represents 1 animal.”

Finally, in Figure 8g, ‘d6’ should read ‘d7’. The correct Figure 8 and its accompanying legend appear below as Figure 1.

**Figure 1:**
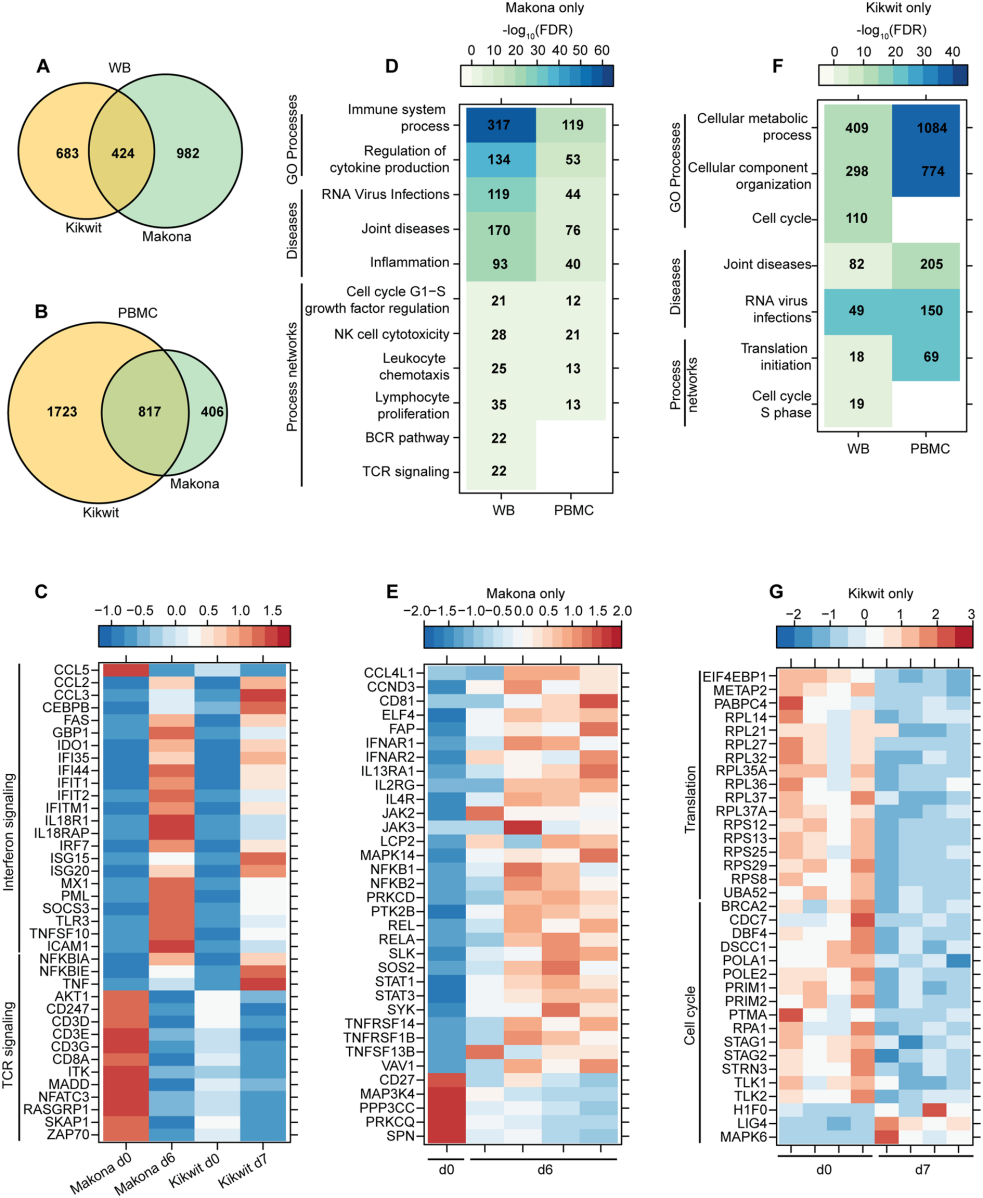
Comparison of host transcriptional profile following ZEBOV-Makona or ZEBOV-Kikwit infection at end stage of disease. (**A**,**B**) Venn diagram shows overlap between DEGs detected following Makona and Kikwit infection 6 and 7 DPI, respectively, in WB **(A)** and PBMC (**B**). (**C**) Heatmap representing gene expression (shown as absolute normalized RPKM values) of shared DEGs detected at end stage of disease following Kikwit or Makona infection that enriched to Process Network terms “Inflammation – Interferon signaling” and “Immune response – TCR signaling”; range of colors is based on scaled and centered RPKM values of the entire set of genes (red represents increased expression while blue represents decreased expression); each column represents the median RPKM values on each day for either Kikwit or Makona infection. (**D**) Heatmap representing functional enrichment of DEGs exclusively detected following Makona infection 6 DPI; color intensity represents the statistical significance (shown as -log_10_ of the FDR-corrected p-Value); range of colors is based on the lowest and highest −log_10_(FDR) values for the entire set of terms; the number of DEGs enriching to each functional enrichment term each day is listed within each box; blank boxes represent no statistical significance. (**E**) Heatmap representing gene expression (shown as absolute normalized RPKM values) of DEGs found only 6 DPI with Makona that enriched to “Lymphocyte proliferation”; day 0 is represented by the median RPKM value, while each column represents 1 animal for 6 DPI. (**F**) Heatmap representing functional enrichment of DEGs exclusively detected following Kikwit infection 7 DPI. (**G**) Heatmap representing gene expression (shown as absolute normalized RPKM values) of DEGs found exclusively 7 DPI following Kikwit infection that enriched to “Translation” and “Cell cycle”; each column represents 1 animal.

