# Supplementary material for: Infection with the Makona variant results in a delayed and distinct host immune response compared to previous Ebola virus variants
Source: Sci Rep. 2017 Aug 29;7:9730. doi: 10.1038/s41598-017-09963-y (PMC5574898; doi:10.1038/s41598-017-09963-y)
Supplement: Supplementary file 1 — Supplementary Information [file 41598_2017_9963_MOESM1_ESM.pdf]

Infection with the Makona variant results in a delayed and distinct host immune response compared to previous Ebola virus variants

Krista Versteeg[1,2¶], Andrea R. Menicucci[3¶], Courtney Woolsey[1,2], Chad E. Mire[1,2], Joan B. Geisbert[1,2], Robert W. Cross[1,2], Krystle N. Agans[1,2], Daniel Jeske[3], Ilhem Messaoudi[3,4*] and Thomas W. Geisbert[1,2*]

Supplementary Figure S1

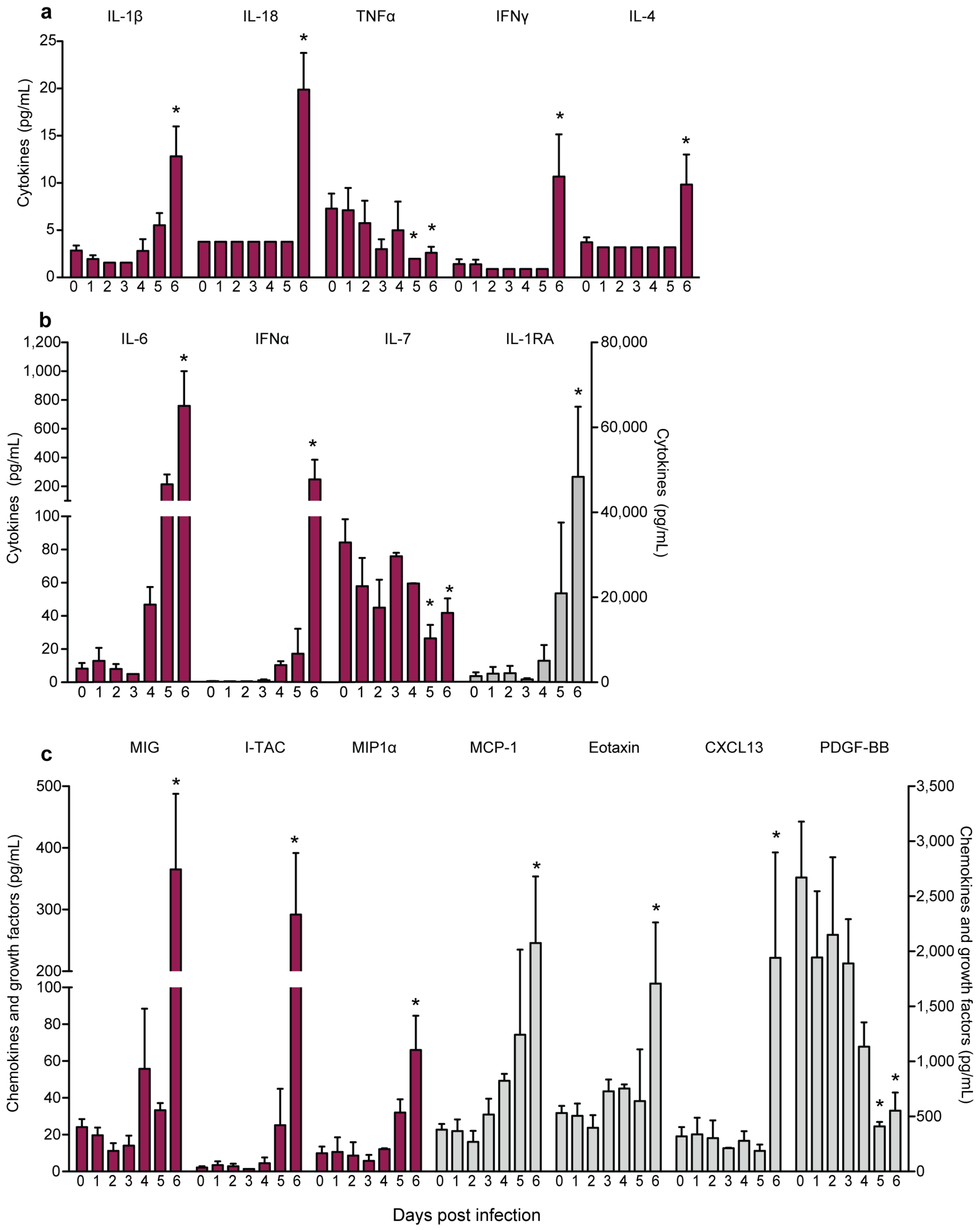

Supplementary Figure S1. Plasma levels of cytokines, chemokines and growth factors. Cytokine **(a-b)**, chemokine and growth factor **(c)** levels in plasma were determined using Luminex technology. Left axis (red bar) and right axis (gray bar). A nonparametric trend where each time point is modeled by its own mean was assumed for statistical analysis; * denote p-value ≤ 0.05 at the indicated time point compared to 0 DPI.

Supplementary Figure S2

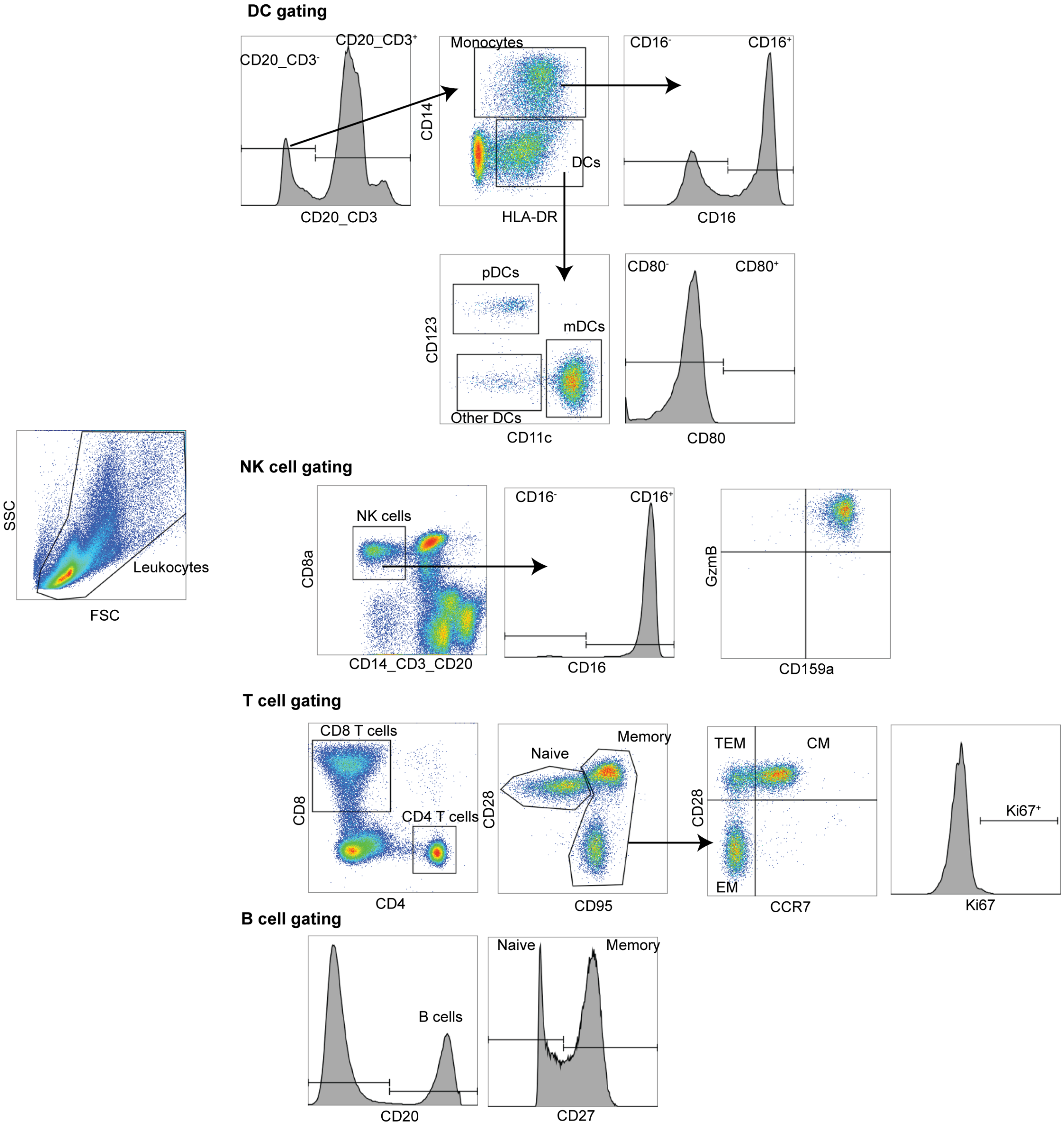

Supplementary Figure S2. Flow cytometry gating strategy. First, lymphocytes expressing CD3 and CD20 were gated out. DC and monocytes were identified based on CD14 and HLA-DR expression. Monocytes were further subdivided based on CD16 expression. DCs were further subdivided into myeloid and plasmacytoid DC based on CD11c and CD123 expression. Activation of DCs was assessed based on CD80 expression levels. NK cells were identified based on absence of CD14, CD3 and CD20 and expression of CD8a. NK cells were further subdivided based on CD16 expression. Activation and effector function of NK cells were monitored by expression of Granzyme B and CD159a. Memory T cells were first identified based on the expression of CD95, and then further subdivided into central (CM), transitional effector (TEM), and effector (EM) memory subsets based on the expression of CD28 and CCR7. T cell proliferation is assessed based on changes in Ki67 expression. Memory B cells were identified based on expression of CD27.

Supplementary Figure S3

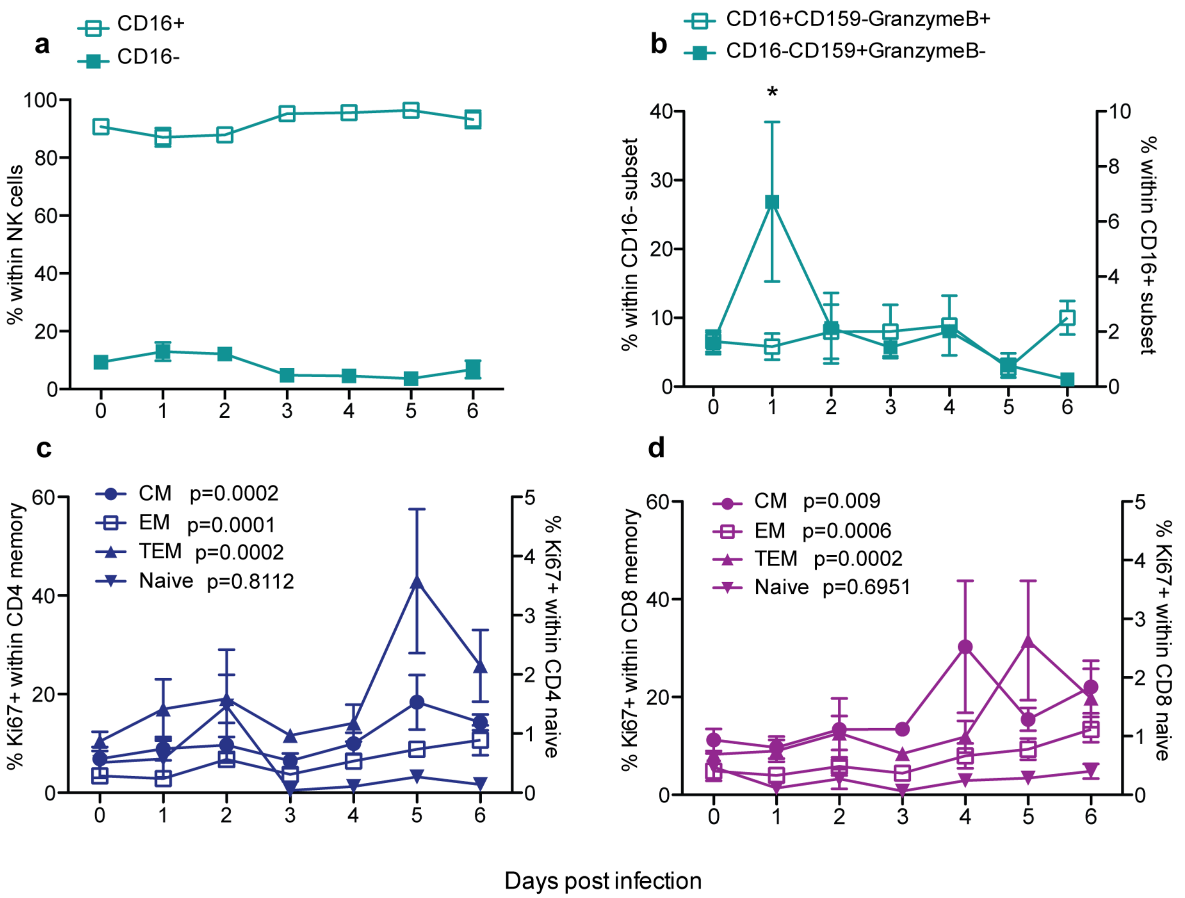

Supplementary Figure S3. NK cell subset frequencies and T cell proliferation. (a) Frequency of $CD16^+$ and $CD16^-$ NK cells was measured by flow cytometry (FCM). **(b)** Frequency of $CD159^-$ $GranzymeB^+$ cells within $CD16^+$ NK cell subset and $CD159a^+GranzymeB^-$ cells within $CD16^-$ NK cell subset. **(c-d)** The magnitude of CD4 T-cell **(c)** and CD8 T-cell **(d)** proliferation was determined by measuring changes in the frequency of $Ki67^+$ cells within naïve, central (CM), effector (EM) and transitional effector (TEM) memory subsets. For frequency changes in T cell subsets, a linear model was used to perform statistical analysis; p-values listed for each parameter represent overall effect throughout infection. For changes in subset frequencies within NK cells, a nonparametric trend where each time point is modeled by its own mean was assumed for statistical analysis; * denote p-value ≤0.05 at the indicated time point compared to 0 DPI.

Supplementary Figure S4

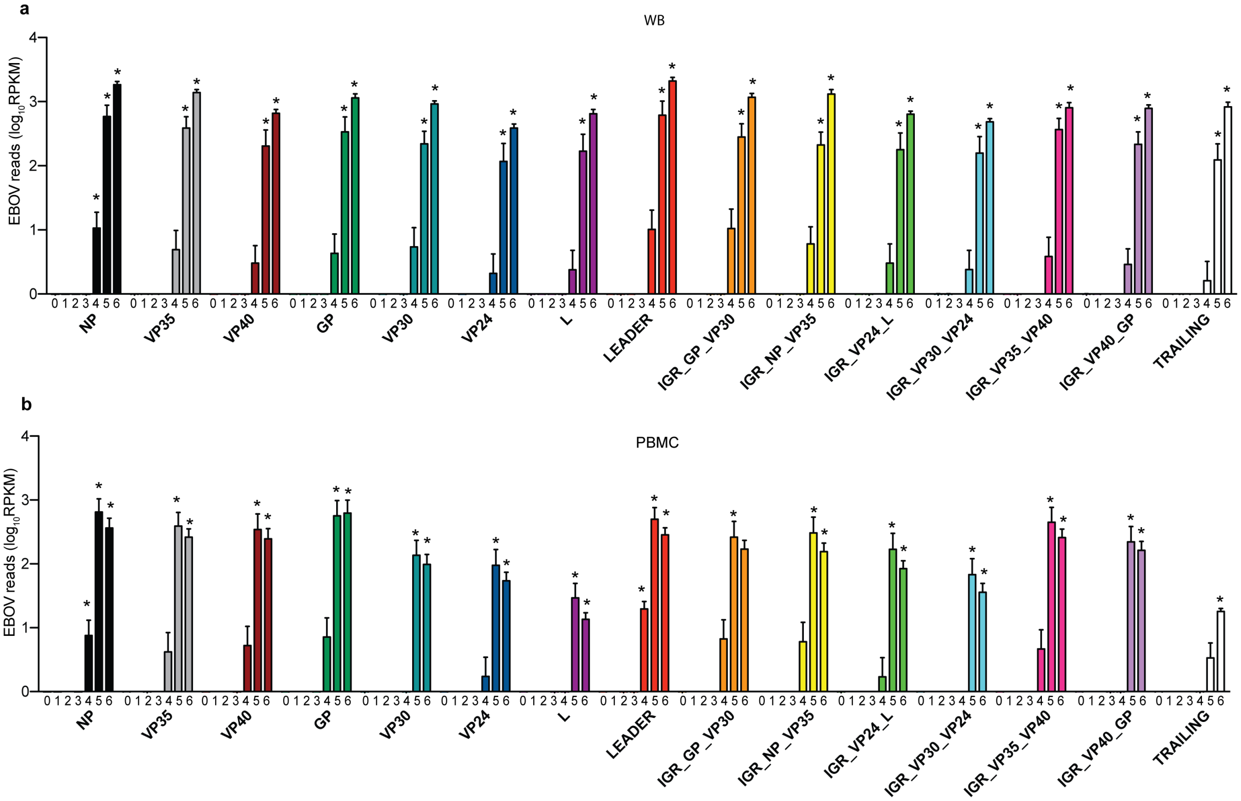

Supplementary Figure S4. EBOV reads mapping to open reading frames and intergenic regions. RPKM normalized transcripts mapping to each EBOV open reading frame (ORF), intergenic region (IGR), as well as leader and trailing sequences at each time point in **(a)** whole blood and **(b)** PBMC. The *EdgeR* package was used to determine statistically significant changes in viral reads; * denote p-value ≤0.05 at the indicated time point compared to 0 DPI.

Supplementary Figure S5

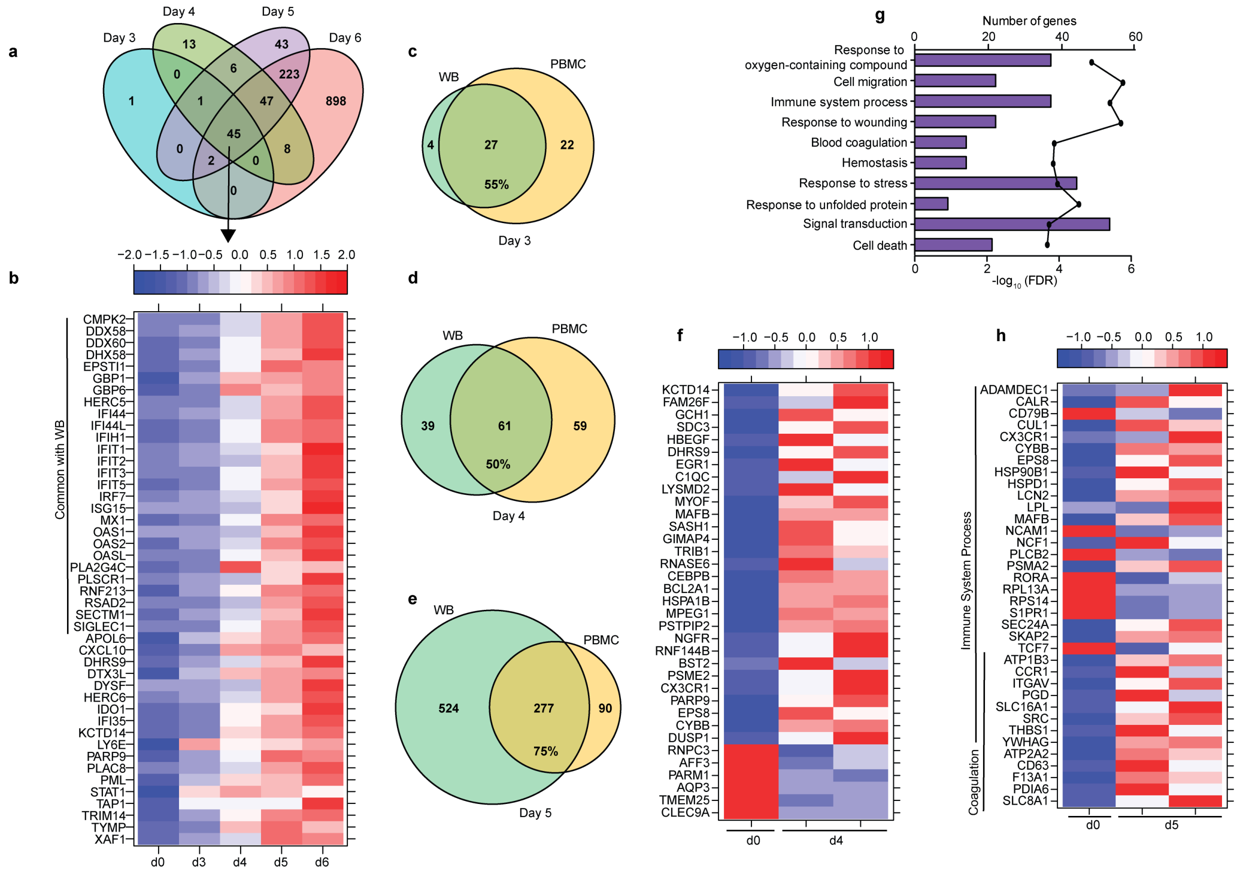

Supplementary Figure S5. Comparison of DEGs detected in PBMC and WB. (a) 4-way venn diagram shows overlap between DEGs detected 3, 4, 5 and 6 DPI. **(b)** Heatmap representing gene expression (shown as absolute normalized RPKM values) of all DEGs upregulated throughout infection; DEGs common with WB are noted; range of colors is based on scaled and centered RPKM values of the entire set of genes (red represents increased expression while blue represents decreased expression); each column represents the median RPKM values for each DPI. **(c-e)** Venn diagram shows overlap between DEGs in PBMC and WB 3 DPI **(c)**, 4 DPI **(d)** and 5 DPI **(e)**. **(f)** Heatmap representing gene expression (shown as absolute normalized RPKM values) of DEGs found exclusively in PBMC 4 DPI with a FC ≥ 3.0; day 0 is represented by the median RPKM value, while each column represents 1 animal for 4 DPI. **(g)** Bar graph depicting statistically significant GO terms to which DEGs found exclusively in PBMC 5 DPI enriched; the line graph represents $-\log_{10}$(FDR) of the enriched term. **(h)** Heatmap representing gene expression (shown as absolute normalized RPKM values) of all DEGs found exclusively in PBMC 5 DPI that enriched to "Immune system process" and/or "Coagulation; day 0 is represented by the median RPKM value, while each column represents 1 animal for 5 DPI.

Supplementary Figure S6

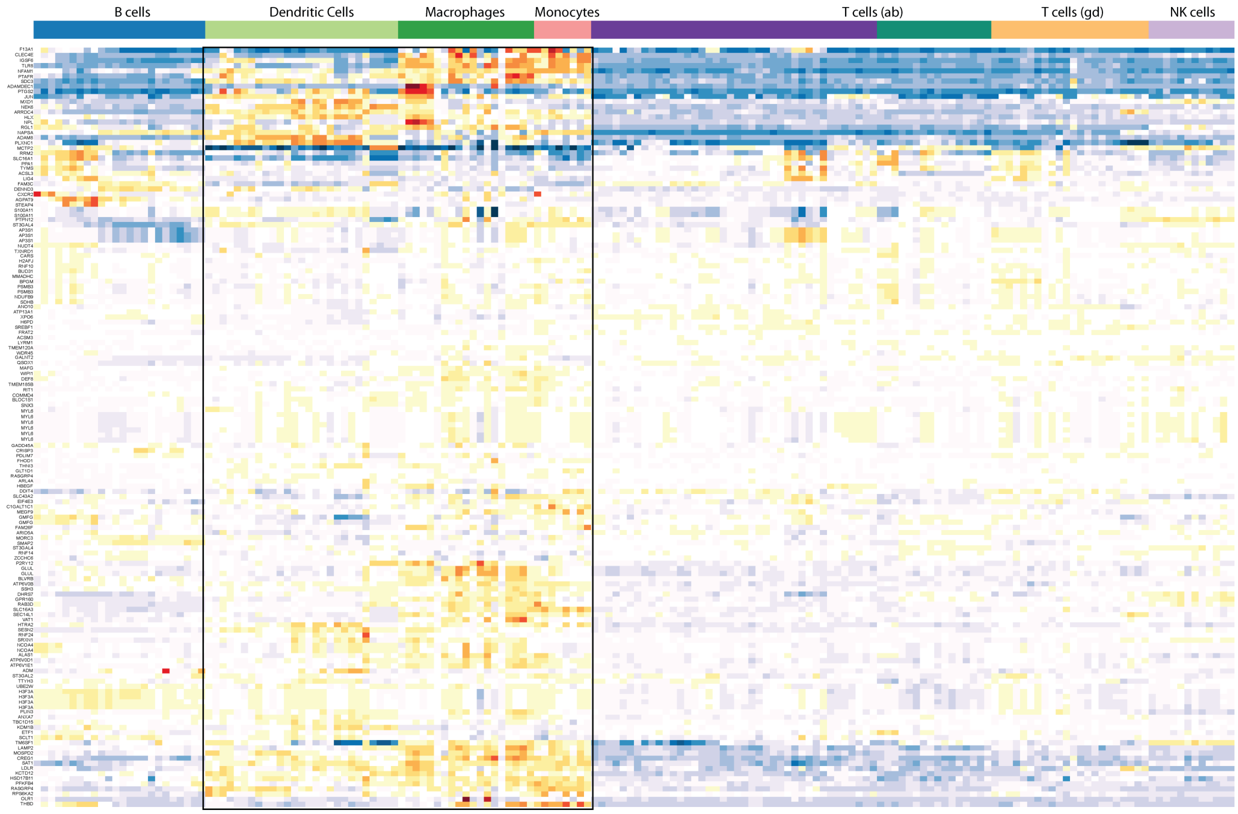

Supplementary Figure S6. Immgen analysis of PBMC 6 DPI. Heatmap profile showing expression profile of DEGs upregulated 6 DPI only in PBMC across various immune cell populations as predicted by ImmGen's MyGeneSet application. Red indicates high while blue indicates low likelihood of expression within indicated immune cell population.

Supplementary Figure S7

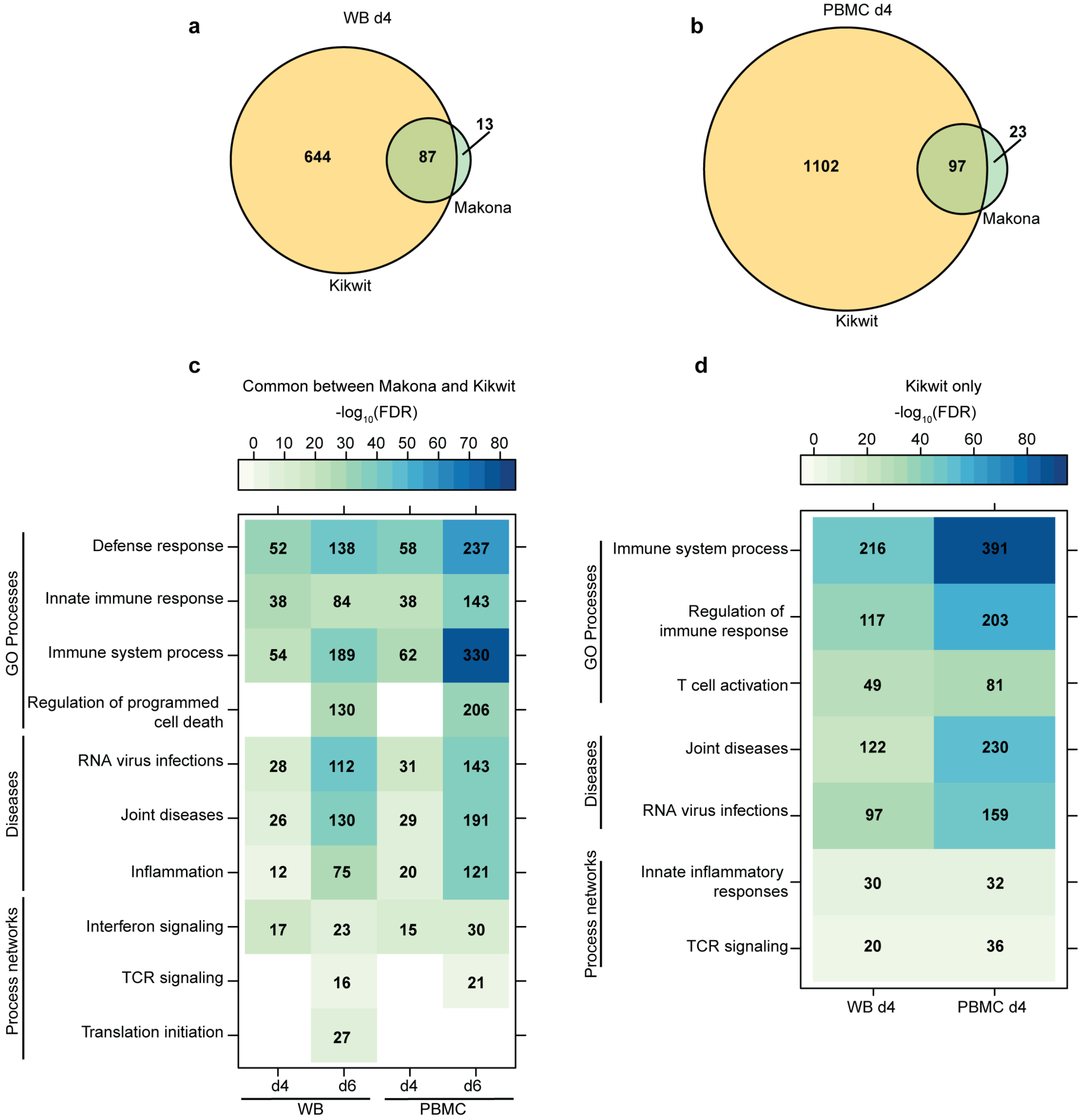

Supplementary Figure S7. Comparison of host transcriptional profile following ZEBOV-Makona or ZEBOV-Kikwit infection at 4DPI. (a-b) Venn diagram shows overlap between DEGs detected following Kikwit and Makona infection 4 DPI in WB **(a)** and PBMC **(b)**. **(c-d)** Heatmap representing functional enrichment of DEGs common following Makona or Kikwit infection 4 and 6 DPI **(c)** and DEGs exclusively detected following Kikwit infection 4 DPI **(d)**; color intensity represents the statistical significance (shown as $-\log_{10}$ of the FDR-corrected p-Value); range of colors are based on the lowest and highest $-\log_{10}$(FDR) value for the entire set of terms; the number of DEGs enriching to each functional enrichment term each day is listed within each box; blank boxes represent no statistical significance.

Supplementary Table S1. Clinical scores used to determine disease progression

Animal	Score	Dyspnea (0-5)	Recumbency (0-9)	Unresponsiveness (0-5)	Bleeding Hemorrhage (0-5)	**Total**
091225	Day 2	0	0	0	0	**0**
100786	Day 2	0	0	0	0	**0**
133465	Day 2	0	0	0	0	**0**
133493	Day 2	0	0	0	0	**0**
0806125	Day 4	0	0	0	0	**0**
1005041	Day 4	0	0	0	0	**0**
903101	Day 6	0	0	1	0	**1**
0906001	Day 6	0	0	1	0	**1**
133440	Day 6	0	0	1	0	**1**
133440	Day 6	0	0	1	1	**2**

Supplementary Table S2. Classification of DEGs detected in WB

**Upregulated**				
**Category**	**Day 3**	**Day 4**	**Day 5**	**Day 6**
**Human homolog**	31	100	779	969
**ncRNA**	0	0	2	6
**uncharacterized**	26	27	106	137
**Total**	**58**	**127**	**885**	**1112**
**Downregulated**				
**Category**	**Day 3**	**Day 4**	**Day 5**	**Day 6**
**Human homolog**	0	0	22	437
**ncRNA**	0	0	0	27
**uncharacterized**	0	0	9	182
**Total**	**0**	**0**	**31**	**646**

Supplementary Table S3. **Antibodies used for flow cytometric analysis of immune cell subsets**

Antibody	Clone	Source
CCR7	G043H7	BioLegend, San Diego, CA
CD3	SP34-2	BD Biosciences, San Jose, CA
CD11c	3.9	BioLegend, San Diego, CA
CD123	6H6	BioLegend, San Diego, CA
CD14	M5E2	BioLegend, San Diego, CA
CD159a	Z199	Beckman Coulter, Brea, CA
CD16	3G8	BioLegend, San Diego, CA
CD20	2H7	BD Biosciences, San Jose, CA
CD27	O323	Tonbo Biosciences, San Diego, CA
CD28	CD28.2	Tonbo Biosciences, San Diego, CA
CD4	OKT4	Tonbo Biosciences, San Diego, CA
CD80	2D10	BioLegend, San Diego, CA
CD8a	RPA-T8	BioLegend, San Diego, CA
CD8b	2ST8.5H7	BD Biosciences, San Jose, CA
CD95	DX2	BD Biosciences, San Jose, CA
Granzyme B	GB11	BioLegend, San Diego, CA
HLA-DR	L243	BioLegend, San Diego, CA
IgG	HP6017	BioLegend, San Diego, CA
Ki67	B56	BD Biosciences, San Jose, CA
